# An efficient synthesis of a C_12_-higher sugar aminoalditol

**DOI:** 10.3762/bjoc.13.213

**Published:** 2017-10-16

**Authors:** Łukasz Szyszka, Anna Osuch-Kwiatkowska, Mykhaylo A Potopnyk, Sławomir Jarosz

**Affiliations:** 1 Institute of Organic Chemistry, Polish Academy of Sciences, Kasprzaka 44/52, 01-224 Warsaw, Poland

**Keywords:** higher carbon sugars, reductive amination, sucrose

## Abstract

The C_12_-aminoalditol H_2_NCH_2_–(CHOBn)_10_–CH_2_OH was prepared from two simple monosaccharide building blocks. The synthesis was realized by a regioselective introduction of the azide group and subsequent protection–deprotection transformations. The chemical reactivity of the aminoalditol was tested in the reductive amination reaction with a selectively protected sucrose monoaldehyde.

## Introduction

**Figure 1:**
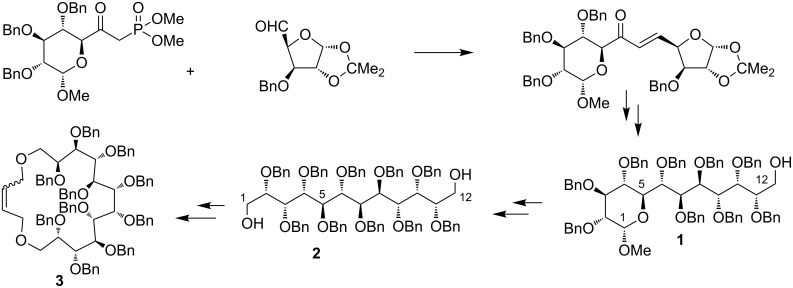
Previous synthesis of a C_12_-higher sugar **1** and its application in the preparation of a polyhydroxylated macrocycle.

Carbohydrates, because of their availability in a variety of optical pure forms, are particularly useful in planning and executing the synthesis of chiral macrocyclic compounds [1–5]. In this context, polyhydroxylated derivatives with long chains are very interesting. However, the synthesis of this kind of molecules is a real challenge in carbohydrate chemistry. Very often reactions, which work well for ‘normal’ (C5–C7) sugars are not applicable for the elongated analogs [6–7]. Recently we have prepared such a derivative by the coupling of two simple monosaccharides and subsequent transformation of the resulting enone into the protected C_12_-sugar **1**. The conversion of the latter into alditol **2** allowed us to prepare derivative **3** having a 18-membered ring (Figure 1) [8].

Higher sugar **1** has a substantial synthetic potential. Besides the approach shown in Figure 1, it could be used in the synthesis of other highly oxygenated targets if the terminal positions at this stage are differentiated. This would allow introducing various functions at either terminal position, thus opening a route to many interesting derivatives, including cyclic ones.

We decided to perform a model study on the efficient differentiation of the terminal positions in such higher sugars or alditols, which could open a route to complex polyhydroxylated derivatives.

## Results and Discussion

A regioselective protection of one of the primary hydroxy groups in diol **2** is not possible. Thus we decided to differentiate the terminal positions in the stage of higher sugar **1**.

**Scheme 1:**
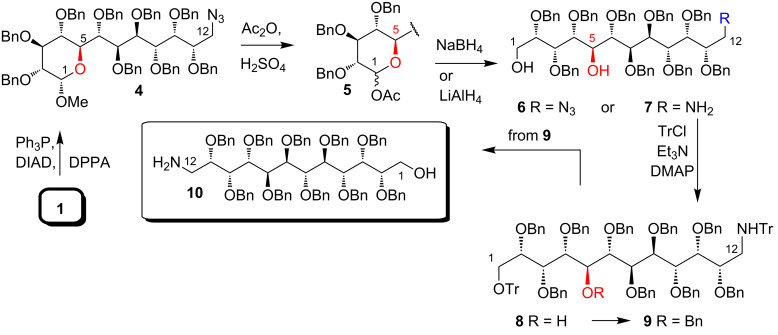
Preparation of C_12_-aminoalditol **10**.

The replacement of the hydroxy group at the C_12_-position with an azide, accomplished successfully under Mistunobu conditions, afforded azide **4** in 95% yield (Scheme 1).

The crucial step was the liberation of the anomeric position (C1), which, as we noticed, can cause problems. We subjected azide **4** to acetolysis and, although this reaction is very capricious in higher sugar chemistry [7–8], succeeded in the preparation of the desired hemiacetal acetate **5** in very good yield (86%) as a mixture of two anomers (α:β = 2:1).

Treatment of azido ester **5** with NaBH_4_ gave predominantly azidodiol **6** (66%) and small amounts of aminodiol **7** (5%). Application of LiAlH_4_ as the reducing agent afforded only compound **7** (Scheme 1). The former result may open, eventually, a new possibility for the preparation of linear higher aminoalditol derivatives.

The subsequent protection of both primary positions in aminodiol **7** with the bulky trityl group (**8**), followed by a protection of the remaining secondary OH group as benzyl ether (**9**) and removal of the temporary trityl group provided aminoalditol **10** (Scheme 1). This compound can be modified selectively at either terminal position: C1 (OH group) or C12 (NH_2_ group).

**Figure 2:**
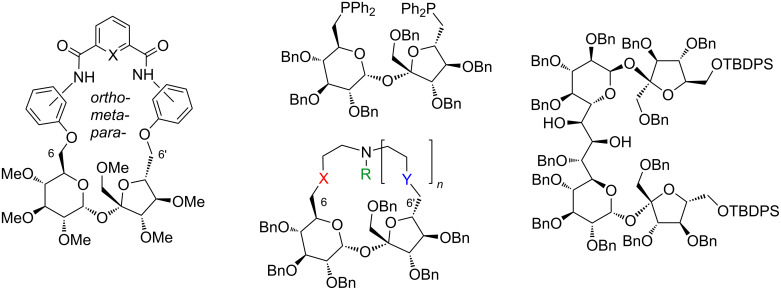
Examples of highly functionalized sucrose derivatives from our laboratory.

Since we are engaged in the preparation of complex derivatives of sucrose (Figure 2) [9–16] we decided to prepare a sucrose derivative with this aminoalditol pendant which could eventually be used in the synthesis of macrocyclic derivatives. Sucrose, undoubtedly the most common disaccharide, has found applications in the synthesis of polymers [17] or surfactants [18]. There is also an increasing interest in the application of sucrose as a ‘normal’ chemical [16,19–20].

**Scheme 2:**
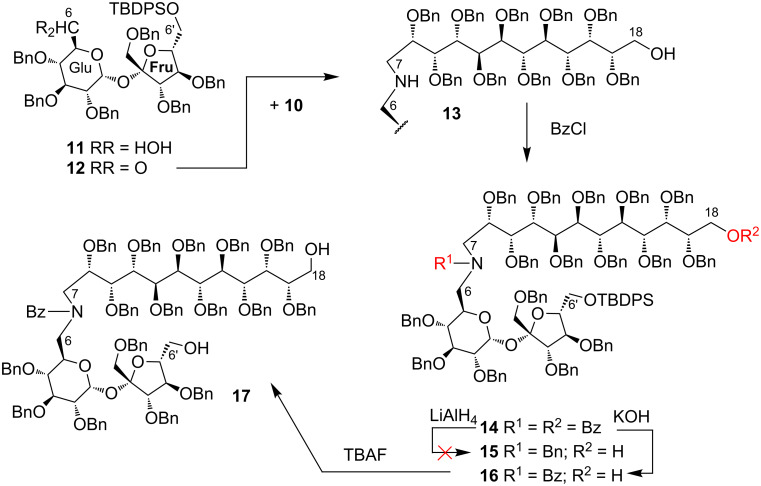
Preparation of a sucrose molecule with a higher aminoalditol pendant.

Our synthesis towards a sucrose derivative with a long alditol pendant was initiated from aldehyde **12** (readily prepared from the known [15] alcohol **11** with the free 6-OH group) and C_12_-aminoalditol **10** (Scheme 2).

Coupling of these two species under the reductive amination conditions provided amine **13** in 78% yield. For the transformation of compound **13** into the terminally functionalized derivative we decided to protect the amino function, located at the C6 position of the sucrose moiety, preferably as benzyl ether **15**. Compound **13** was, therefore, treated with benzoyl chloride which installed the benzoyl group at the amino and the free hydroxy functions providing dibenzoyl derivative **14** in very good yield (94%, Scheme 2).

The reduction of derivative **14** with LiAlH_4_ removed the benzoyl protection from both hydroxy and amino groups and surprisingly, no reduction of the benzoyl located at the amino or the benzyl groups was noted.

Therefore, compound **14** was treated with 1 M KOH to remove the benzoyl groups only from the hydroxy group, which yielded compound **16**, the starting material in the next step of our synthesis. Subsequent removal of the *tert*-butyldiphenylsilyl group from the C6’ position with TBAF afforded **17** in 93% yield (Scheme 2). Amide **16** with the free OH group at the C18 position and **17** having two hydroxy groups at the C18 and C6’ positions can be regarded as convenient starting materials for the preparation of more complex sucrose derivatives.

## Conclusion

In summary, we have developed an efficient method for the synthesis of a C_12_-aminoalditol H_2_NCH_2_**–**(CHOBn)_10_**–**CH_2_OH **10**. The chemical reactivity of this valuable synthon was demonstrated by its high yielding coupling with the C-6 position of a selectively protected sucrose by a reductive amination reaction.

## Experimental

### General

NMR spectra were recorded using Varian 600 MHz or Bruker 500 MHz spectrometers in CDCl_3_ at 25 °C unless otherwise stated. The structures were assigned, whenever necessary, with the help of 2D correlation experiments (COSY, HSQC, HMBC). Chemical shifts (in most cases only diagnostic signals were shown) were reported with reference to TMS. Optical rotations were measured with a Jasco P 1020 polarimeter (sodium light) in chloroform at 20 °C. The MS spectra were recorded with a Mariner PerSeptive Biosystems spectrometer. Thin-layer chromatography was performed on pre-coated plates (0.25 mm, silica gel 60 F_254_). Column chromatography was carried out with silica gel (230–400 mesh).

### Synthesis of azide **4**

This reaction was conducted under an argon atmosphere. To a cooled (to 0 °C) solution of alcohol **1** [8] (0.89 g, 0.75 mmol) and Ph_3_P (0.4 g, 1.5 mmol) in dry THF (12 mL), DIAD (0.3 mL, 1.5 mmol) was added and the mixture was stirred for 10 min. Diphenylphosphoryl azide (DPPA, 0.32 mL, 1.48 mmol) was then added dropwise within 10 min, the mixture was allowed to the reach 20 °C, and stirring was prolonged for another 1.5 h. Ethyl acetate (10 mL) was added and the mixture was washed with 1 M HCl (10 mL). The organic phase was separated and the aqueous phase extracted with ethyl acetate (2 × 10 mL). The combined organic solutions were washed with aqueous saturated NaHCO_3_ (15 mL) and brine (15 mL), dried, concentrated, and the product was isolated by column chromatography (hexanes/ethyl acetate 8:1) to afford pure compound **4** (0.87 g, 0.7 mmol, 95%) as an oil. [α]_D_ +12.1 (*c* 0.4); ^1^H NMR (600 MHz) δ 7.41–7.11 (m, 35H, ArH), 4.91 (d, *J =* 11.0, 1H, benzylic H), 4.80 (d, *J* = 11.5 Hz, 1H, benzylic H), 4.79 (d, *J* = 11.2 Hz, 1H, benzylic H), 4.80–4.67 (m, 4H, benzylic H), 4.64 (d, *J* = 11.6 Hz, 2H, benzylic H), 4.62 (d, *J* = 11.8 Hz, 2H, benzylic H), 4.61 (d, *J* = 12.2 Hz, 1H, benzylic H), 4.59 (d, *J*_1,2_ = 3.5 Hz, 1H, H-1), 4.53 (d, *J* = 11.5 Hz, 1H , benzylic H), 4.49 (d, *J* = 11.5 Hz, 1H, benzylic H), 4.42 (s, 1H, benzylic H), 4.41 (s, 1H, benzylic H), 4.39 (d, *J* = 11.5, 1H, benzylic H), 4.28 (d, *J* = 11.7 Hz, 1H, benzylic H), 4.22 (d, *J*_5,4_ = 10.3 Hz, 1H, H-5), 4.18–4.13 (m, 2H, H-7, H-8), 4.05 (dd, *J*_9,8_ = 5.8 Hz, *J*_9,10_ = 4.3 Hz, 1H, H-9), 4.01 (d, *J*_6,7_ = 9.8 Hz, 1H, H-6), 3.97 (dd, *J*_3,4_ = 9.2 Hz, *J*_3,2_ = 9.3 Hz, 1H, H-3), 3.83 (dd, *J*_4,5_ = 10.1 Hz, 1H, H-4), 3.75 (m, 1H, H-10), 3.67 (m, 1H, H-11), 3.40 (dd, 1H, H-2), 3.29 (s, 3H, OCH_3_), 3.26–3.17 (m, 2H, H-12, H-12’) ppm; ^13^C NMR (150 MHz) δ 138.86, 138,86, 138.81, 138.75, 138.72, 138.69, 138.23, 138.12, 138.01 (9 × C_quat_-Ph), 130.05 (C-Ph), 128.42–127.05 (m, 41 × C-Ph), 126.0 (C-Ph), 120.24 (C-Ph), 120.21 (C-Ph), 97.84 (C-1), 82.79 (C-3), 80.05 (C-2), 79.71 (C-10), 79.18 (C-11), 78.55 (C-4), 78.45 (C-6), 77.54 (C-7), 77.34 (C-8), 77.11 (C-9), 75.39, 74.65, 74.36, 73.33, 73.29, 73.01, 72.38, 72.08, 71.73 (9 × O*C*H_2_Ph), 69.64 (C-5), 55.01 (OCH_3_), 51.83 (C-12) ppm; HRMS (ESI) *m*/*z*: [M + Na]^+^ calcd for C_76_H_79_N_3_O_11_Na, 1232.5590; found, 1232.5612; Anal. calcd for C_76_H_79_N_3_O_11_: C, 75.41; H, 6.58; N, 3.47; found: C, 75.41; H, 6.47; N, 3.34.

### Acetolysis of glycoside **4**; synthesis of **5**

Azide **4** (0.36 g, 0.3 mmol) was dissolved in ethyl acetate (2.3 mL) to which acetic anhydride (4.6 mL) and sulfuric acid (0.72 mL of the solution: conc. H_2_SO_4_ (0.05 mL) in ethyl acetate (100 mL)) were added. The mixture was stirred at 20 °C for 46 h and then diluted with ether (15 mL). It was then neutralized with saturated aq NaHCO_3_ (15 mL), the organic phase was separated, and the aqueous phase extracted with ether (2 × 10 mL). The combined organic solutions were washed with water (40 mL) and brine (40 mL), dried, concentrated, and the crude product was purified by column chromatography (hexanes/ethyl acetate 9:1) to yield **5** (0.32 g, 0.26 mmol, 86%) as a 2:1 mixture of α/β anomers (calculated by integration of the anomeric signals at δ: 6.34 and 5.63). ^1^H NMR (600 MHz) δ 7.32–7.13 (m, 35H, ArH), 6.34 (d, *J*_1,2_ = 3.6 Hz, 1H, H-1), 4.88 (d, *J* = 11.0 Hz, 1H, benzylic H), 4.85–4.37 (m, 43H, benzylic H), 4.34 (dd, *J*_5,4_ = 10 Hz, *J*_5,6_ = 2.4 Hz, 1H, H-5), 4.27 (d, *J* = 11.9 Hz, 1H, benzylic H), 4.16–4.05 (m, 3H, H-7, H-8, H-9), 3.93–3.86 (m, 2H, H-3, H-4), 3.73 (dd, 1H, H-10), 3.69–3.62 (m, 2H, H-6, H-11), 3.47 (dd, *J*_2,3_ = 9.0 Hz, 1H, H-2), 3.23–3.22 (d, 2H, H-12, H-12’), 1.97 (s, 3H, CH_3_) ppm; ^13^C NMR (150 MHz) δ 169.50 (C=O), 138.95, 138.65, 138.62, 138.60, 138.55, 138.46, 138.13, 137.99, 137.61 (9 × C_quat_-Ph), 128.44–127.08 (m, 45 × C-Ph), 89.58 (C-1), 85.26 (C-6), 82.26 (C-3), 79.62 (C-10), 79.06 (C-2), 78.90 (C-11), 77.79 (C-4), 77.63, 77.39, 77.34 (C-7, C-8, C-9), 75.32, 74.70, 74.54, 73.16, 73.10, 72.84, 72.61, 72.22, 72.14 (9 × O*C*H_2_Ph), 72.61 (C-5), 51.78 (C-12), 20.94 (CH_3_) ppm; HRMS (ESI) *m*/*z*: [M + Na]^+^ calcd for C_77_H_79_N_3_O_12_Na: 1260.5537; found, 1260.5561; Anal. calcd for C_77_H_79_N_3_O_12_: C, 74.61; H, 6.51; N, 3.39; found: C, 74.70; H, 6.58; N, 3.38.

### Preparation of compounds **6** and **7**

**Method a:** To a solution of **5** (0.23 g, 0.18 mmol) in THF/methanol (3:1 v/v, 30 mL), NaBH_4_ (3 g, 79 mmol) was added in few portions and the mixture was stirred for 3 h at 20 °C. Then it was partitioned between ether (30 mL) and water (15 mL), the organic phase was separated, and the aqueous phase extracted with ether (2 × 15 mL). The combined organic solutions were washed with water (10 mL), dried, concentrated, and the residue was subjected to column chromatography (hexanes/ethyl acetate 5:1 to 3:1) to give two products: **6** (0.15 g, 0.12 mmol, 66%) and **7** (12 mg, 5%). Products **6** and **7** were isolated by preparative TLC (for **6** hexanes/ethyl acetate 2:1, for **7** CH_2_Cl_2_/MeOH 10:1).

Product **6**: [α]_D_ −6.5 (*c* 0.5); ^1^H NMR (600 MHz) δ 7.29–7.15 (m, 45H, 45 × H-Ph), 4.77 (d, *J* = 11.5 Hz 1H,), 4.70–4.44 (m, 19H), 4.35 (m, 1H, H-5), 4.23–4.16 (m, 2H), 4.02–3.94 (m, 3H), 3.87–3.82 (m, 2H), 3.80 (dd, *J* = 3.7 Hz, *J* = 5.9 Hz, 1H), 3.71 (dd, *J* = 4.3 Hz, *J* = 10.3 Hz, 1H), 3.48 (dd, *J*_1,2_ = 4.5 Hz, *J*_1,1’_ = 12.0 Hz, 1H, H-1), 3.39 (dd, *J*_12,11_ = 3.1 Hz, *J*_12,12’_ = 12.8 Hz, 1H, H-12), 3.33–3.26 (m, 2H, H-1’, H-12’) ppm; ^13^C NMR (150 MHz) δ 128.42–127.25 (m, 45 × C-Ph), 79.78, 79.74, 79.35, 79.15, 79.12, 78.87, 78.68, 77.76, 77.67, 74.69, 74.03, 73.74, 73.47, 73.16, 72.82, 72.73, 72.04, 72.04 (9 × O*C*H_2_Ph), 70.74 (C-5), 61.60 (C-1), 51.88 (C-12) ppm; HRMS (ESI) *m*/*z*: [M + Na]^+^ calcd for C_75_H_79_N_3_O_11_Na, 1220.5627; found, 1220.5612; Anal. calcd for C_75_H_79_N_3_O_11_: C, 75.16; H, 6.64; N, 3.51; found: C, 75.13; H, 6.82; N, 3.40.

**Method b:** To a solution of **5** (0.3 g, 0.24 mmol) in dry THF (20 mL), LiAlH_4_ (55 mg, 1.45 mmol) was slowly added and the mixture was stirred for 1 h at 20 °C. Excess hydride was carefully decomposed with aqueous saturated Na_2_SO_4_ (5 mL). Celite (0.5 g) was added, the mixture was stirred for 5 min, and then filtered through a short pad of silica which was subsequently washed with ethyl acetate (3 × 20 mL). The organic solution was dried and concentrated to give **7** (0.27 g) as a single product, which was used in the next step without purification. The structure of **7** was confirmed by MS. MS (ESI) *m*/*z*: [M + H]^+^ calcd for C_75_H_81_NO_11_, 1172.6; found, 1172.8.

### Synthesis of aminoalcohol **10**

Crude compound **7** was dissolved in dichloromethane (20 mL) containing triethylamine (0.4 mL), DMAP (5 mg) and trityl chloride (0.4 g, 1.4 mmol). The mixture was boiled under reflux for 24 h, then cooled to 20 °C, and partitioned between ether (20 mL) and water (20 mL). The layers were separated and the aqueous layer extracted with ether (2 × 20 mL). The combined organic solutions were washed with water (20 mL) and brine (20 mL), dried, concentrated, and the residue was purified by column chromatography (hexanes/ethyl acetate 100:0 to 90:10) to yield alcohol **8** (0.19 g, 0.11 mmol, 47% after two steps). [α]_D_ +1.0 (*c* 0.2); ^1^H NMR (600 MHz) δ 7.4–6.90 (m, 75H, 75 × H-Ph), 4.74–4.58 (m, 7H, 7 × benzylic H), 4.56 (d, *J* = 11.7 Hz, 1H, benzylic H), 4.50 (d, *J* = 11.2 Hz, 1H, benzylic H), 4.49 (d, *J* = 11.3 Hz, 1H, benzylic H), 4.48 (d, *J* = 11.2 Hz, 1H, benzylic H), 4.45 (d, *J* = 11.4 Hz, 1H, benzylic H), 4.41–4.32 (m, 4H, 4 × benzylic H), 4.31–4.21 (m, 4H, H-6, H-5, H-9, benzylic H), 4.16 (d, *J* = 11.6 Hz, 1H, benzylic H), 4.06–4.03 (m, 2H, H-8, H-7), 4.00–3.98 (m, 2H, H-4, H-10), 3.94–3.89 (m, 2H, H-2, H-11), 3.78 (m, 1H, H-3), 3.46 (br s, 1H, NH), 3.35 (dd *J*_1,1’_ = 10.0 Hz, *J*_1,2_ = 6.3 Hz, 1H, H-1), 3.25 (dd, 1H, H-1’), 2.44 (dd, *J*_12,12’_ = 12.4 Hz, *J*_12,11_ = 3.9 Hz, 1H, H-12), 2.37 (dd, 1H, H-12’) ppm; ^13^C NMR (150 MHz) δ 145.92 (3C, 3 × C_quat_-Ph), 143.95 (3C, 3 × C_quat_-Ph), 139.34, 139,10, 139.01, 138.97, 138.57, 138.54, 138.24, 138.16, 137.83 (9 × C_quat_-Ph), 128.71–126.07 (m, 75 × C-Ph), 86.96 (O*C*Ph_3_), 80.36 (C-11), 80.21 (C-4), 80.01 (C-8), 78.24 (C-3), 78.11 (C-9), 77.83 (C-6), 77.72 (C-7), 77.69 (C-2), 76.7 (C-10), 74.52, 73.06, 72.97, 72.86, 72.61, 72.42, 72.34, 72.34, 71.69 (9 × O*C*H_2_Ph), 71.58 (C-5), 70.67 (N*C*Ph_3_), 63.64 (C-1), 43.62 (C-12) ppm; HRMS (ESI) *m*/*z*: [M + H]^+^ calcd for C_113_H_110_NO_11_: 1656.8102; found: 1656.8079; Anal. calcd for C_113_H_109_NO_11_: C, 81.90; H, 6.63; N, 0.85; found: C, 81.92; H, 6.72; N, 0.88.

To a stirred solution of **8** (0.35 g, 0.21 mmol) in DMF (20 mL) containing imidazole (1.5 mg), sodium hydride (60% dispersion in mineral oil, 80.6 mg, 2.1 mmol) was added, and the mixture was stirred at 20 °C for 30 min. Benzyl bromide (0.2 mL, 1.68 mmol) was added dropwise and the mixture was stirred for 24 h at 20 °C. Excess of hydride was decomposed by careful addition of water (5 mL) and the mixture was partitioned between water (30 mL) and ether (40 mL). The layers were separated and the aqueous one extracted with ethyl acetate (3 × 20 mL). The combined organic solutions were washed with water (40 mL) and brine (40 mL), dried, concentrated, and the crude **9** (0.4 g) was used to the next step without purification.

Crude compound **9** was dissolved in ether/methanol (v/v 1:1; 40 mL) to which *p-*TsOH∙H_2_O (3.5 g, 18 mmol) was added, and the mixture was stirred and boiled under reflux for 24 h. Then it was cooled to 20 °C and partitioned between ethyl acetate (50 mL) and water (50 mL). The organic phase was separated and the aqueous layer was extracted with ethyl acetate (2 × 30 mL). The combined organic solutions were washed with water (50 mL), aq saturated Na_2_CO_3_ (30 mL), and brine (30 mL), dried, concentrated, and the product was isolated by column chromatography (hexanes/ethyl acetate 5:1 to 1:2) to afford **10** (0.24 g, 0.19 mmol, 87% after two steps). [α]_D_ −15.7 (*c* 0.2); ^1^H NMR (600 MHz) δ 7.35–7.10 (m, 50H, 50 × H-Ph), 4.91 (d, *J* = 11.0 Hz, 1H), 4.90 (d, *J* = 11.9 Hz, 1H), 4.78 (d, *J* = 11.0 Hz, 1H), 4.74 (d, *J* = 11.9 Hz, 1H), 4.67 (d, *J* = 11.0 Hz, 1H), 4.67 (d, *J* = 11.0 Hz, 1H), 4.63 (d, *J* = 11.9 Hz, 1H), 4.54–4.42 (m, 13H), 4.36 (d, *J* = 11.2 Hz, 1H), 4.26–4.18 (m, 7H), 4.02 (m, 1H), 3.91 (dd, *J* = 5.0 Hz, *J* = 5.3 Hz, 1H), 3.75 (dd, *J* = 5.6 Hz, *J* = 5.2 Hz, 1H), 3.59 (m, 2H), 3.52 (dd, *J* = 4.7 Hz, *J* = 11.9 Hz, 1H), 3.30 (dd, *J* = 3.6 Hz, *J* = 11.9 Hz, 1H), 2.74 (dd, *J* = 4.5 Hz, *J* = 13.7 Hz, 1H), 2.64 (dd, *J* = 5.6 Hz, *J* = 13.7 Hz, 1H) ppm; ^13^C NMR (150 MHz) δ 139.42, 139.05, 139.01, 138.68, 138.59, 138.59, 138.40, 138.32, 138.32, 138.18 (10 × C_quat_-Ph), 128.34–127.17 (m, 50 × C-Ph), 81.66, 81.01, 80.52, 79.99, 79.11, 78.97, 79.97, 78.80, 78.19, 78.19, 75.24, 74.98, 74.74, 74.63, 73.71, 72.52, 72.44, 72.23, 71.86, 71.75 (10 × O*C*H_2_Ph), 61.46 (C-1), 42.09 (C-12) ppm; HRMS (ESI) *m*/*z*: [M + H]^+^ calcd for C_82_H_88_NO_11_, 1262.6356; found, 1262.6357.

### Synthesis of sucrose with a long chain at the C6-position; preparation of **17**

This reaction was conducted under an argon atmosphere. Sucrose alcohol **11** [10] (110 mg, 0.1 mmol) was oxidized under Swern conditions [21] to afford aldehyde **12**. To a solution of this crude aldehyde in CH_2_Cl_2_ (5 mL), freshly dried molecular sieves (150 mg), acetic acid (6 μL, 0.1mmol), and aminoalcohol **10** (130 mg, 0.1 mmol in 5 mL CH_2_Cl_2_) were added, and the mixture was stirred at 20 °C for 2 h. NaBH_3_CN (7.5 mg, 0.12 mmol) was added and stirring was continued overnight at rt. Water (20 mL), 25% NH_3_ aq (0.5 mL), and CH_2_Cl_2_ (10 mL) were added, the phases were separated, and the aqueous layer was extracted with CH_2_Cl_2_ (3 × 20 mL). The combined organic solutions were washed with water (30 mL) and brine (30 mL), dried, concentrated, and the crude material was purified by flash chromatography (hexanes/ethyl acetate 5:1 to 2:1) to afford **13** (0.19 g, 0.08 mmol, 78%). [α]_D_ −5.3 (*c* 0.2); ^1^H NMR (500 MHz) δ 7.59–7.61, 7.28–7.04 (m, 90H, 90 × H-Ph), 5.68 (d, *J* = 3.5 Hz, 1H, H-1), 4.80 (d, *J* = 11.3 Hz, 1H), 4.78 (d, *J* = 11.0 Hz, 1H), 4.73 (d, *J* = 11.0 Hz, 1H), 4.69 (d, *J* = 11.5 Hz, 1H), 4.67–4.15 (m, 34H), 4.09–4.02 (m, 2H), 4.03 (d, *J* = 11.6 Hz, 1H), 3.95 (d, *J* = 10.7 Hz, 1H), 3.94 (d, *J* = 10.7 Hz, 1H), 3.93–3.85 (m, 2H), 3.83 (d, *J* = 10.4 Hz, 1H), 3.82 (d, *J* = 10.5 Hz, 1H), 3.81–3.74 (m, 2H), 3.56–3.48 (m, 3H), 3.42 (d, *J* = 11.7 Hz, 1H), 3.30 (dd, *J* = 3.5 Hz, *J* = 9.6 Hz, 1H), 3.20 (d, *J* = 9.7 Hz, 1H), 2.78 (dd, *J* = 3.3 Hz, *J* = 12.5 Hz, 1H), 2.65 (dd, *J* = 6.7 Hz, *J* = 12.6 Hz, 1H), 2.61–2.51 (m, 2H), 1.90 (s, 1H), 1.00 (s, 9H, *t*-Bu) ppm; ^13^C NMR (125 MHz) δ 139. 37, 139.16, 139.12, 138.95, 138.89, 138.89, 138.85, 138.82, 138.60, 138.54, 138.37, 138.35, 138.34, 138.29, 138.29, 137.94, 133.41, 133.22 (18 × C_quat_-Ph), 135.57, 135.49, 129.64, 129.61, 128.30–127.08 (m) (90 × C-Ph), 105.13 (C-2’), 90.47 (C-1), 84.25, 83.67, 81.77, 81.68, 81.19, 81.17, 80.68, 80.33, 80.23, 80.23, 79.14, 79.01, 79.01, 78.43, 78.14, 78.03, 70.61, (C-2, C-3, C-3’ C-4, C-4’ C-5, C-5’, C-8, C-9, C-10, C-11, C-12, C-13, C-14, C-15, C-16, C-17), 75.36, 74.83, 74.76, 74.65, 74.38, 74.33, 73.46, 73.35, 72.97, 72.57, 72.47, 72.30, 72.13, 72.04, 71.84, 71.62, 70.44 (16 × O*C*H_2_Ph, C-1’), 65.37, 61.57 (C-6’, C-18), 51.41 (N*C*H_2_), 49.78 (N*C*H_2_), 26.92 (triple intensity, 3C – *t*-Bu), 19.24 (C_quat_, *t*-Bu) ppm; HRMS (ESI) *m*/*z*: [M + Na]^+^ calcd for C_152_H_161_NO_21_SiNa, 2388.1334; found, 2388.1295; Anal. calcd for C_152_H_161_NO_21_Si: C, 77.16; H, 6.86; N, 0.59; found: C, 77.13; H, 6.78; N, 0.57.

To a solution of **13** (150 mg, 0.063 mmol) in dry dichloromethane (5 mL), containing DMAP (1 mg), and triethylamine (53 μL, 0.38 mmol), benzoyl chloride (30 μL, 0.25 mmol) was added dropwise, and the mixture was stirred for 5 h at 20 °C. It was then partitioned between water (5 mL) and saturated aqueous NaHCO_3_ (2 mL), the layers were separated, and the aqueous one extracted with dichloromethane (2 × 15 mL). The combined organic solutions were dried, concentrated, and the residue was purified by column chromatography (hexanes/ethyl acetate 5:1 to 2:1) to afford **14** (0.15 g, 0.06 mmol, 94%). [α]_D_ −11.0 (*c* 0.2); ^1^H NMR (500 MHz, C_7_D_8_, 80 °C) δ 7.75–7.72 (m, 2H, H-Ph), 7.55–7.46 (m, 4H, H-Ph), 7.24–7.16 (m, 3H, H-Ph), 7.12–6.64 (m, 91H, 91 × H-Ph), 5.60 (d, *J* = 2.3 Hz, 1H, H-1), 4.81–3.59 (m, 58H), 3.52 (d, *J* = 11.1 Hz, 1H), 0.88 (s, 9H, *t*-Bu) ppm; ^13^C NMR (125 MHz, C_7_D_8_, 80 °C) δ 135.68, 135.56, 132.37, 132.32, 129.63, 129.56, 128.77, 128.47–126.93 (m), 125.00 (100 × C-Ph), 90.54 (C-1), 84.76, 84.16, 83.14, 82.69, 81.32, 80.59, 80.44, 80.34, 80.16, 80.02, 79.79, 79.55, 79.15, 78.93, 78.61, 78.38, 78.25 (C-2, C-3, C-3’ C-4, C-4’ C-5, C-5’, C-8, C-9, C-10, C-11, C-12, C-13, C-14, C-15, C-16, C-17), 75.07, 74.72, 74.58, 74.54, 74.18, 73.92, 73.45, 73.39, 73.25, 72.93, 72.69, 72.58, 72.40, 71.99, 71.86, 71.53, 71.29 (16 × O*C*H_2_Ph, C-1’), 70.07, 69.91 (C-6’, C-18), 67.05, 65.43 (2 × NCH_2_), 26.82 (triple intensity, 3C – *t*-Bu) ppm; HRMS (ESI) *m*/*z*: [M + 2Na]^+^ calcd for C_166_H_169_NO_23_SiNa_2_, 2618.1662; found, 2618.1639; Anal. calcd for C_166_H_169_NO_23_Si: C, 77.45; H, 6.62; N, 0.54; found: C, 77.22; H, 6.70; N, 0.51.

To a solution of **14** (30 mg, 0.01 mmol) in dry THF (4 mL), LiAlH_4_ (2 mg, 0.044 mmol) was added, the mixture was stirred for 5 h at 20 °C and then boiled under reflux for additional 5 h. After cooling to rt, the excess of hydride was decomposed with aq saturated Na_2_SO_4_ (0.5 mL). Celite (100 mg) was added, the mixture was stirred for 5 min and then was filtered through a short pad of silica which was subsequently washed with ethyl acetate (3 × 10 mL). The organic solution was dried and concentrated to give **13** (15 mg) as a single product. HRMS (ESI) *m*/*z*: [M + Na]^+^ calcd for C_152_H_161_NO_21_SiNa, 2388.1334; found, 2388.1295.

Compound **14** (108 mg, 0.042 mmol) was dissolved in dry THF (2 mL) to which methanol (2 mL) and 1 M KOH (0.5 mL) were added, and the mixture was stirred at 20 °C overnight. The mixture was neutralized with 1 M HCl and partitioned between ethyl acetate (10 mL) and water (10 mL). The aqueous layer was extracted with ethyl acetate (2 × 10 mL) and the combined organic solutions were washed with water (10 mL) and brine (10 mL), dried, and concentrated. The crude material was purified by column chromatography (hexanes/ethyl acetate 6:1 to 3:1) to give product **16** (86.3 mg) detected by MS (HRMS (ESI) *m*/*z*: [M + 2Na]^+^ calcd for C_159_H_165_NO_22_SiNa_2_, 2514.1377; found, 2514.1384) and traces of secondary amine **13**.

To a solution of the above crude mixture (43.6 mg) in THF (4 mL) tetrabutylammonium fluoride trihydrate (35 mg) was added and the mixture was stirred overnight at 20 °C. Then it was concentrated and the residue was purified by preparative thin-layer chromatography (hexanes/acetone 1:1) to yield pure compound **17** (36.7 mg, 0.016 mmol, 93%). [α]_D_ −9.5 (*c* 0.5); ^1^H NMR (500 MHz, C_7_D_8_, 80 °C) δ 7.54–7.43 (m, 4H, H-Ph), 7.40–6.97 (m, 81H, H-Ph), 5.65 (d, *J* = 3.4 Hz, H-1, 1H), 5.12–3.55 (m, 57H) ppm; ^13^C NMR (125 MHz, C_7_D_8_, 80 °C) δ 128.63, 128.48, 128.20–126.80, 124.86 (85 × C-Ph), 91.04 (C-1), 84.95, 84.78, 83.01, 82.97, 82.41, 82.15, 81.31, 81.17, 80.99, 80.93, 80.66, 80.37, 80.25, 80.06, 79.92, 79.79, 79.22 (C-2, C-3, C-3’ C-4, C-4’ C-5, C-5’, C-8, C-9, C-10, C-11, C-12, C-13, C-14, C-15, C-16, C-17), 75.40, 74.90, 74.58, 74.53, 74.24, 73.96, 73.83, 73.75, 73.51, 72.83, 72.59, 72.42, 72.35, 71.97, 71.54, 71.01, 70.96 (16 × O*C*H_2_Ph, C-1’), 66.32, 61.92 (C-6’, C-18) ppm; HRMS (ESI) *m*/*z*: [M + 2Na]^+^ calcd for C_143_H_147_NO_22_Na_2_, 2276.0198; found, 2276.0199; Anal. calcd for C_143_H_147_NO_22_: C, 76.96; H, 6.64; N, 0.63; found: C, 76.85; H, 6.59; N, 0.64.

## Supporting Information

File 1 Copies of NMR spectra.
