# Supplementary material for: An efficient synthesis of a C12-higher sugar aminoalditol
Source: Beilstein J Org Chem. 2017 Oct 16;13:2146–52. doi: 10.3762/bjoc.13.213 (PMC5669227; doi:10.3762/bjoc.13.213)
Supplement: File 1 — Copies of NMR spectra. [file Beilstein_J_Org_Chem-13-2146-s001.pdf]

Supporting information

for

An efficient synthesis of a C_{12}-higher sugar aminoalditol

Łukasz Szyszka, Anna Osuch-Kwiatkowska, Mykhaylo A. Potopnyk, and Sławomir Jarosz*

Address: Institute of Organic Chemistry, Polish Academy of Sciences, Kasprzaka 44/52, 01-224 Warsaw, Poland

Email: Sławomir Jarosz - slawomir.jarosz@icho.edu.pl

* Corresponding author

Copies of NMR spectra

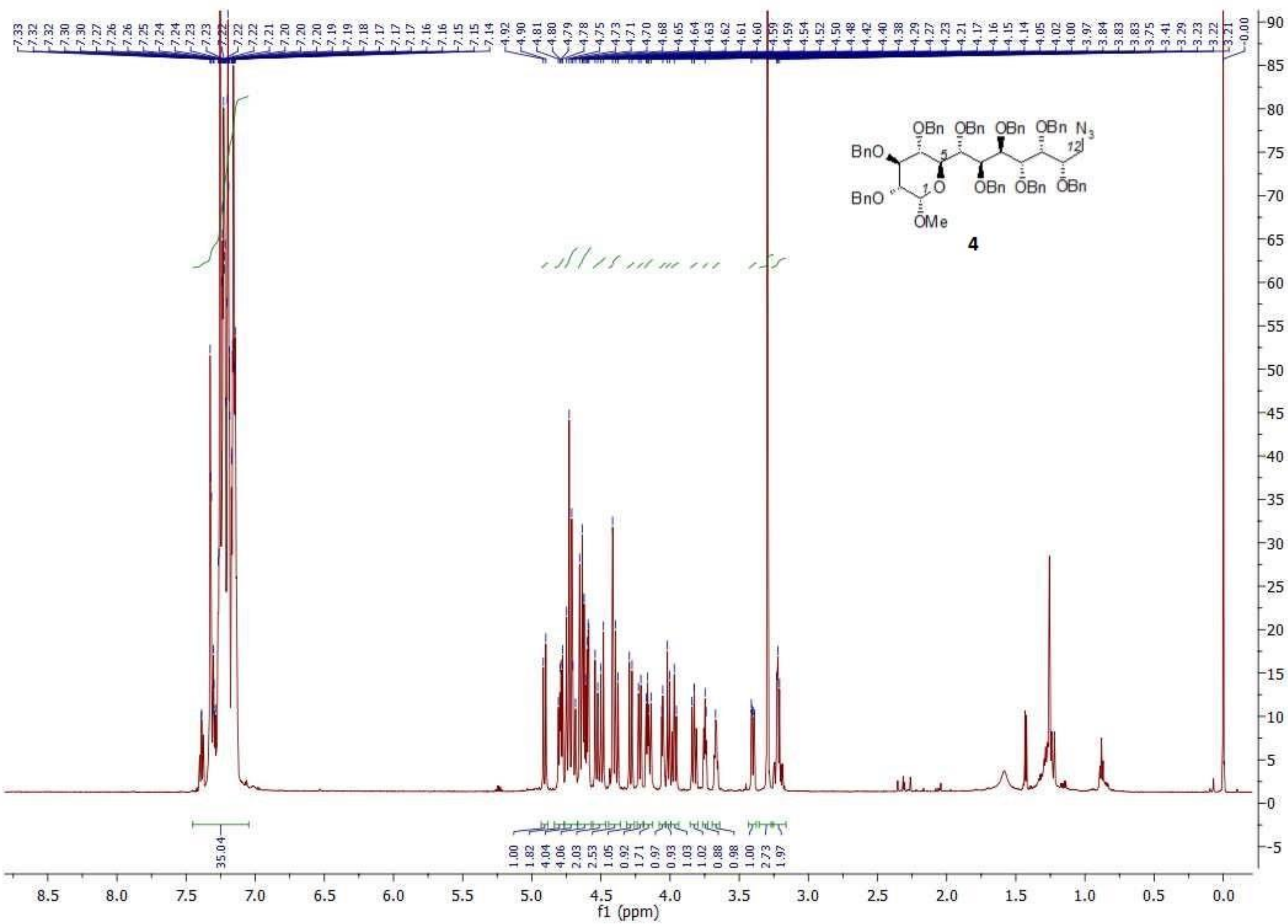

Figure S1: ^{1}H NMR spectrum of compound **4**.

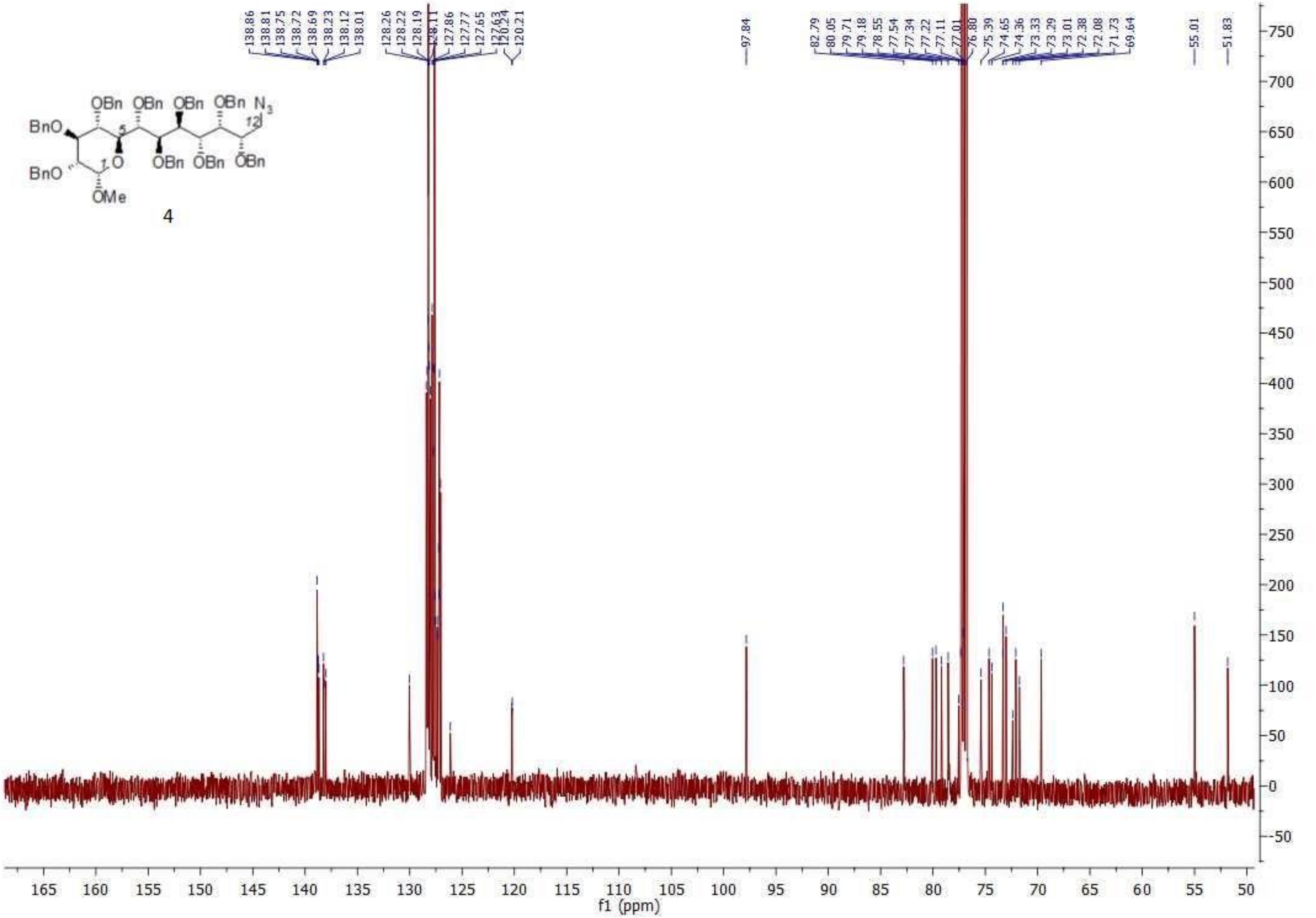

Figure S2: ^{13}C NMR spectrum of compound **4**.

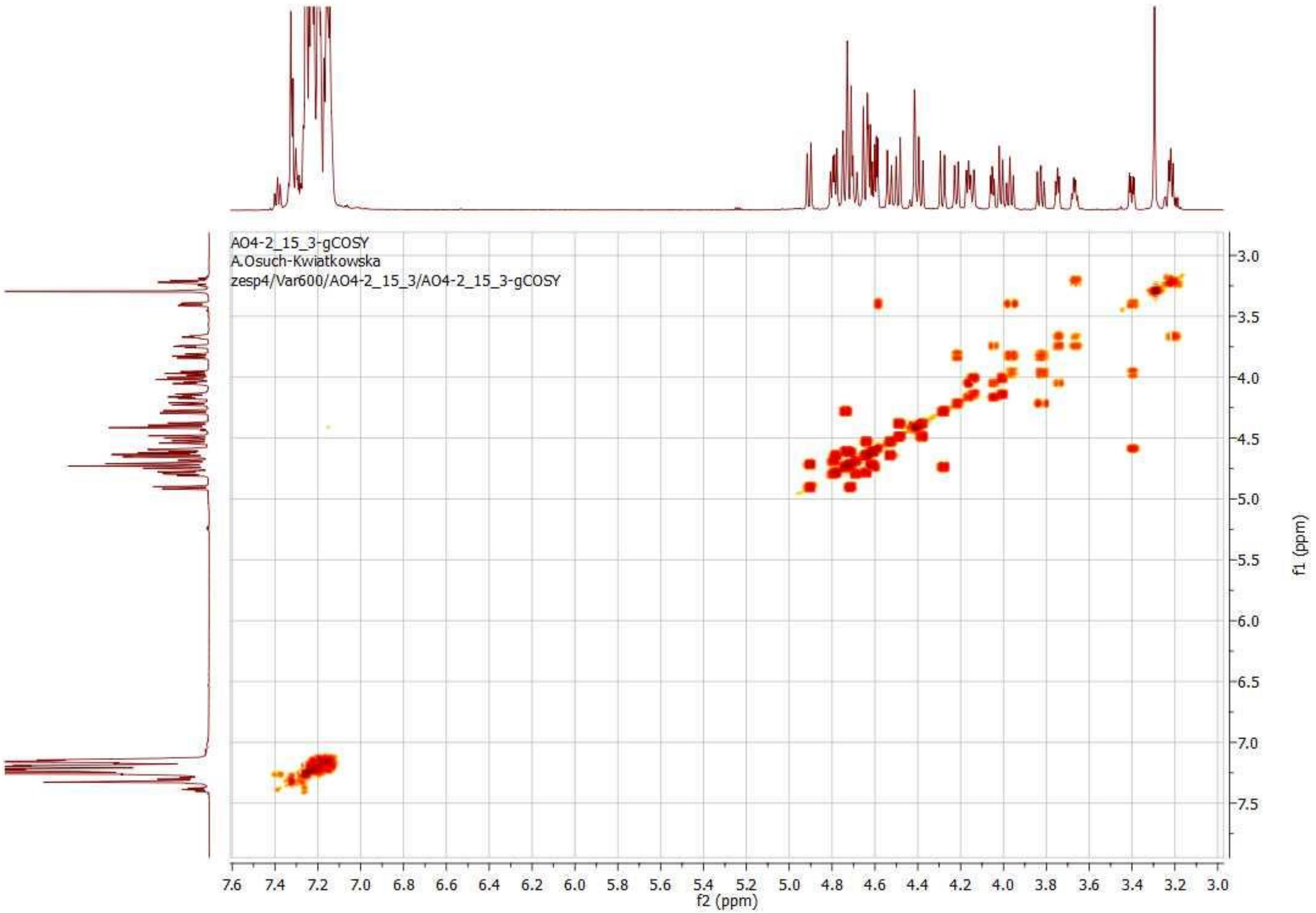

Figure S3: gCOSY spectrum of compound **4**.

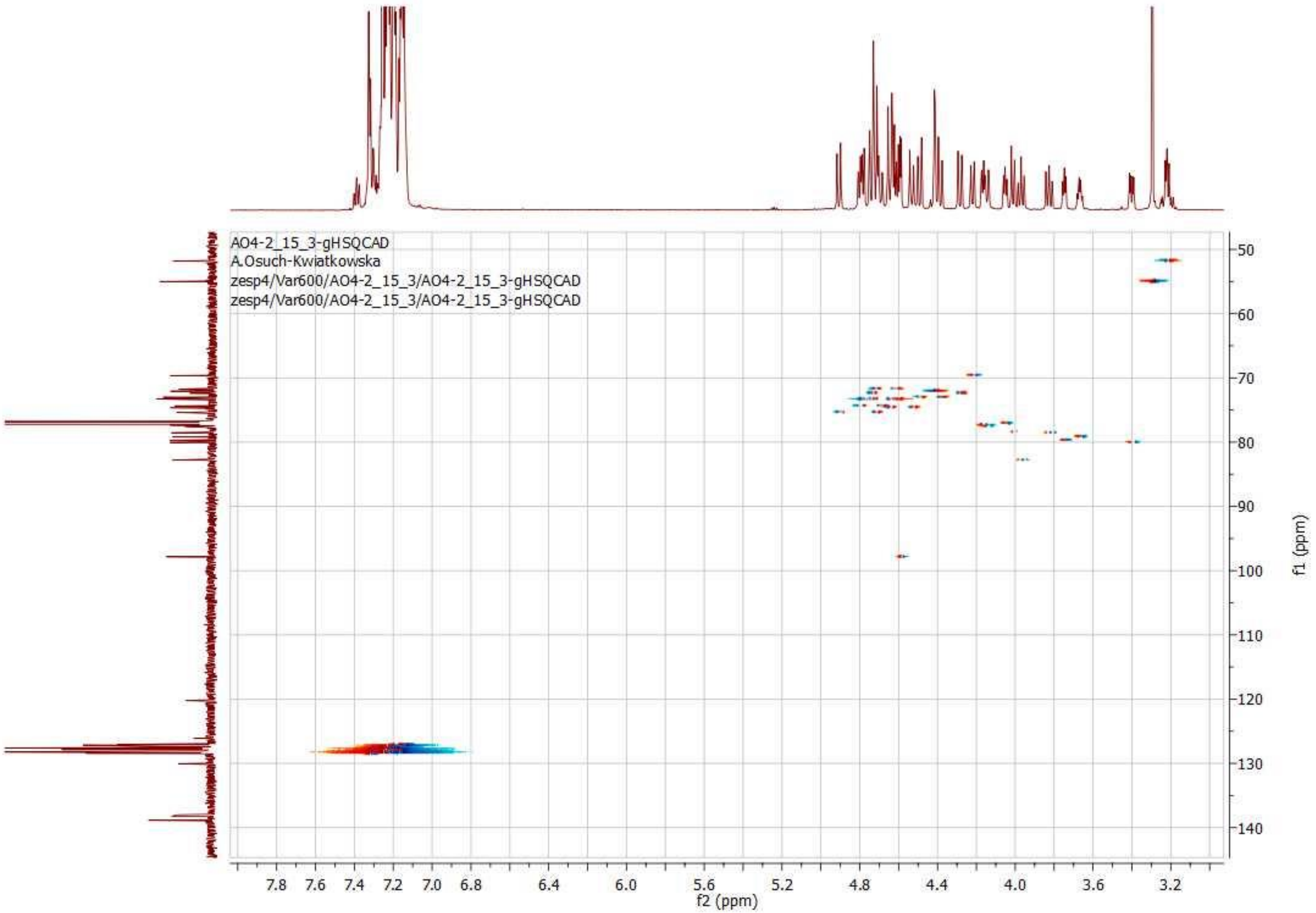

Figure S4: gHSQCAD spectrum of compound **4**.

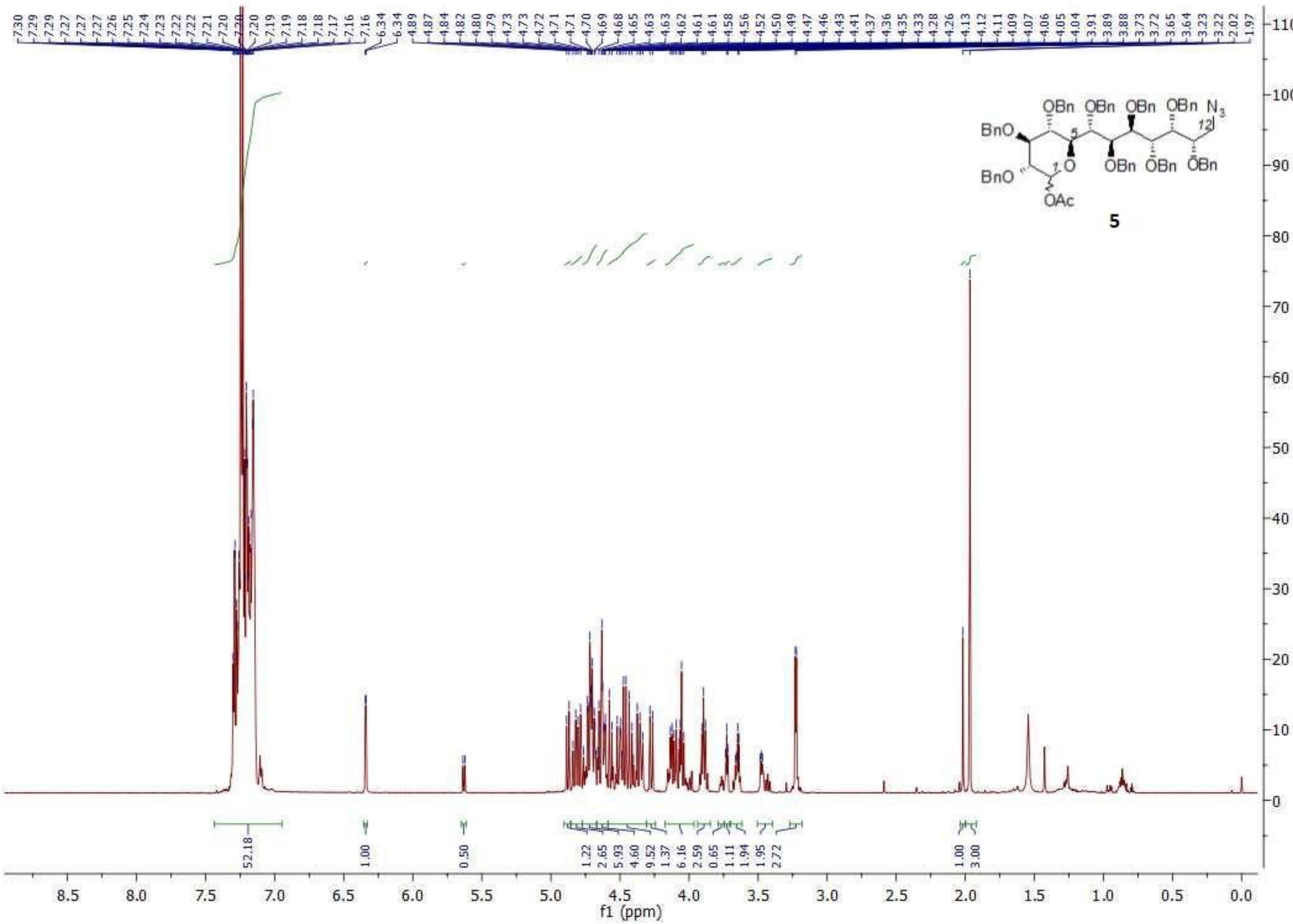

Figure S5: ^{1}H NMR spectrum of compound **5**.

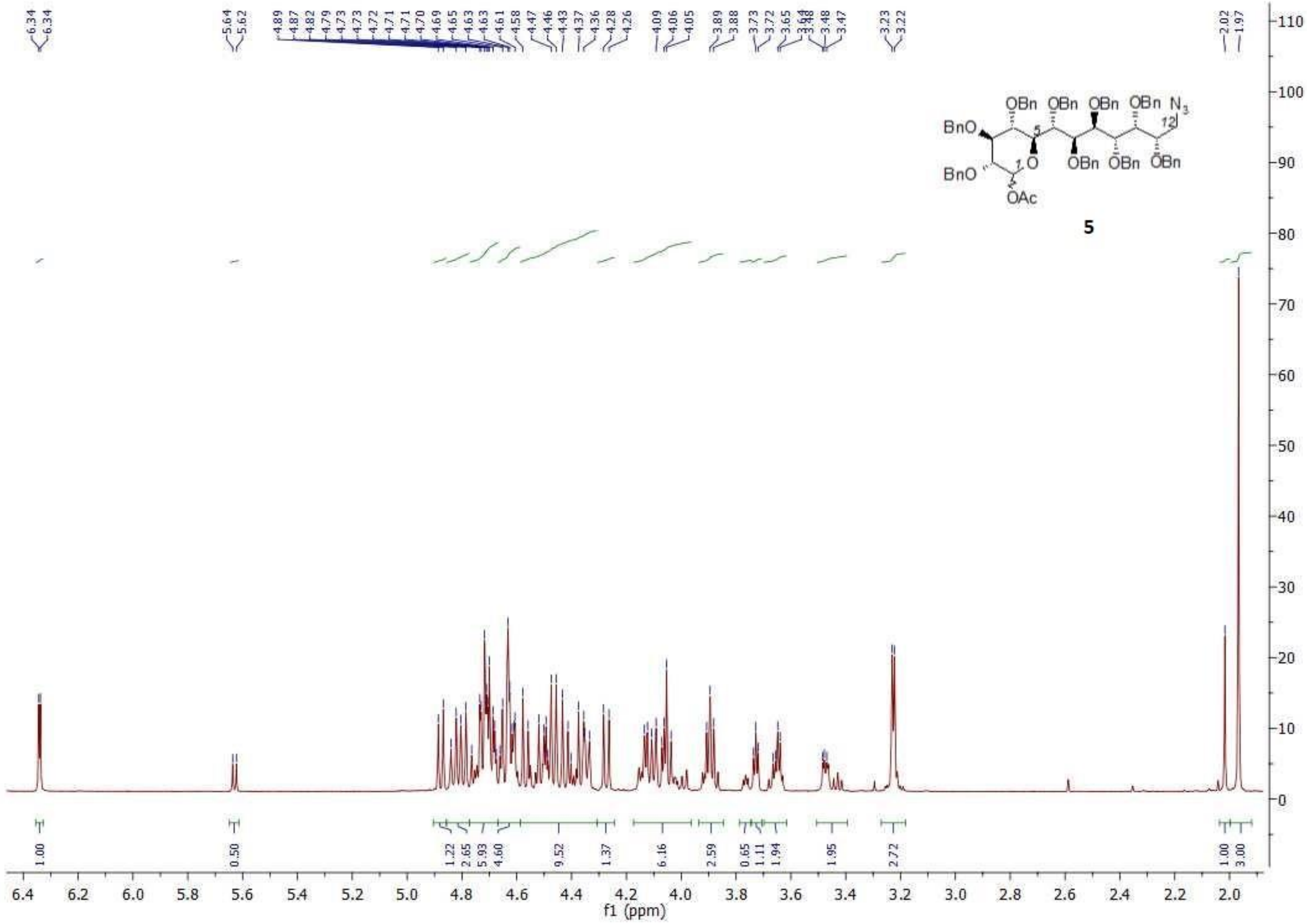

Figure S6: ^{1}H NMR spectrum (aliphatic part) of compound **5.**

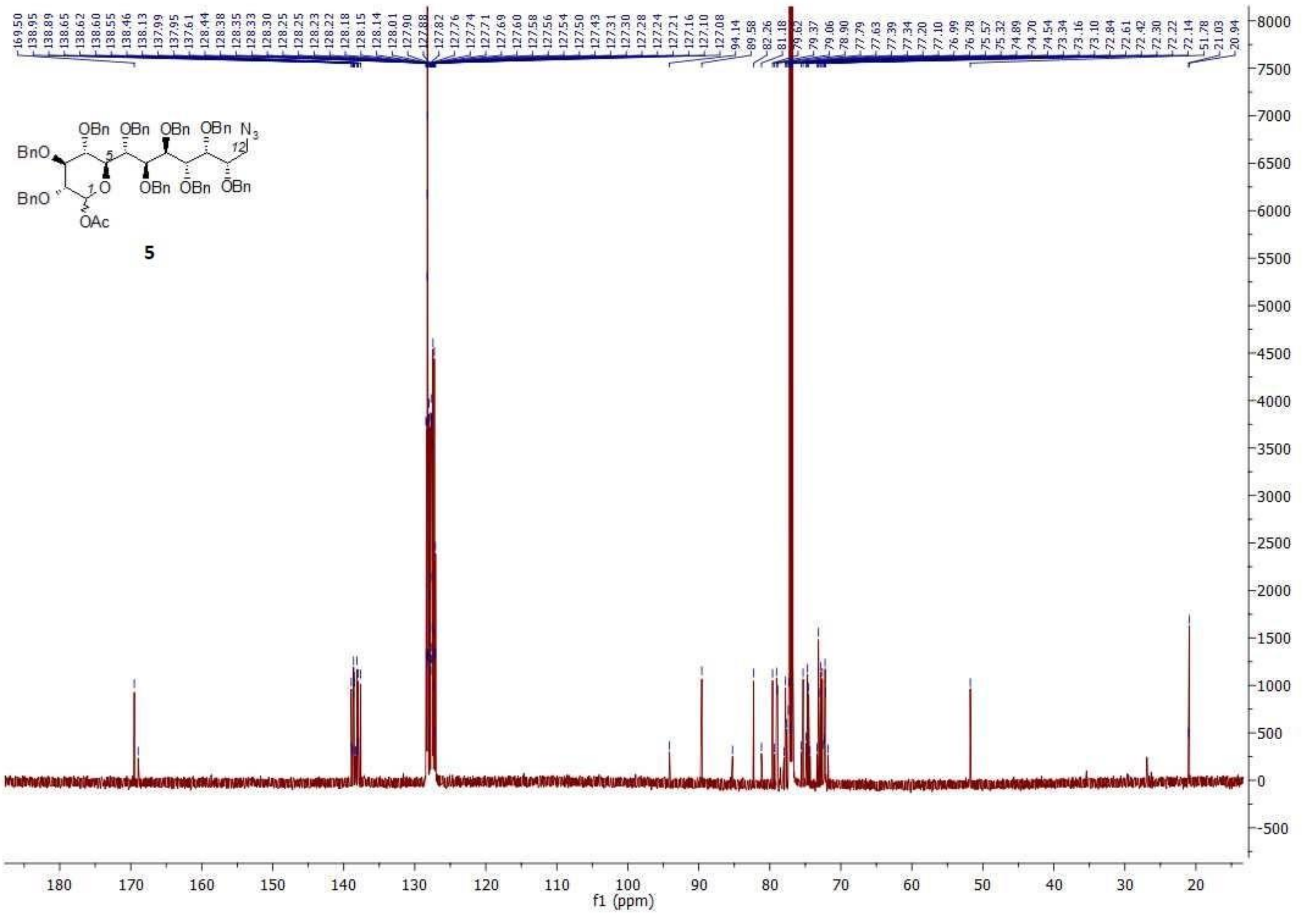

Figure S7: ^{13}C NMR spectrum of compound **5**.

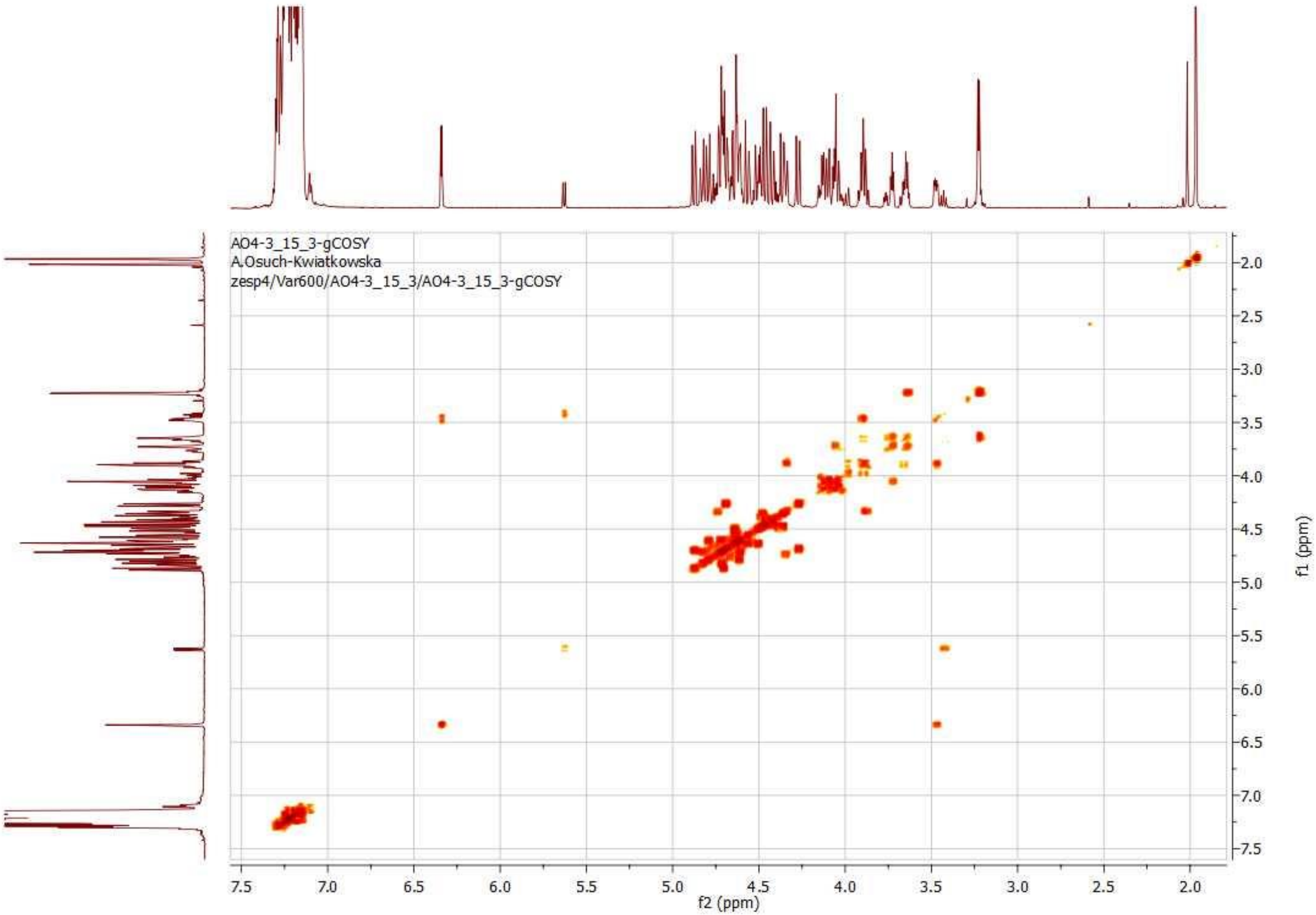

Figure S8: gCOSY spectrum of compound **5**.

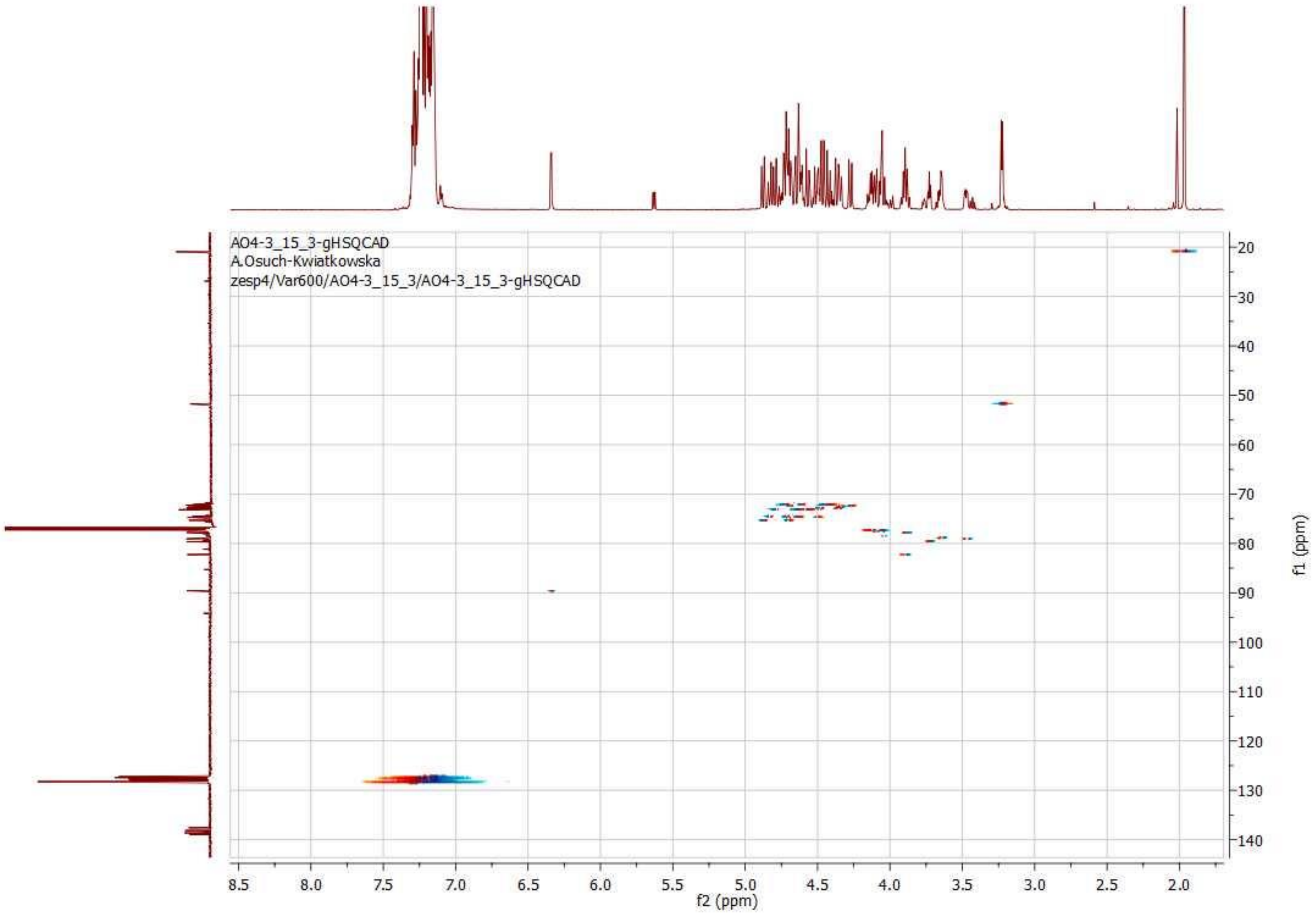

Figure S9: gHSQCAD spectrum of compound **5**.

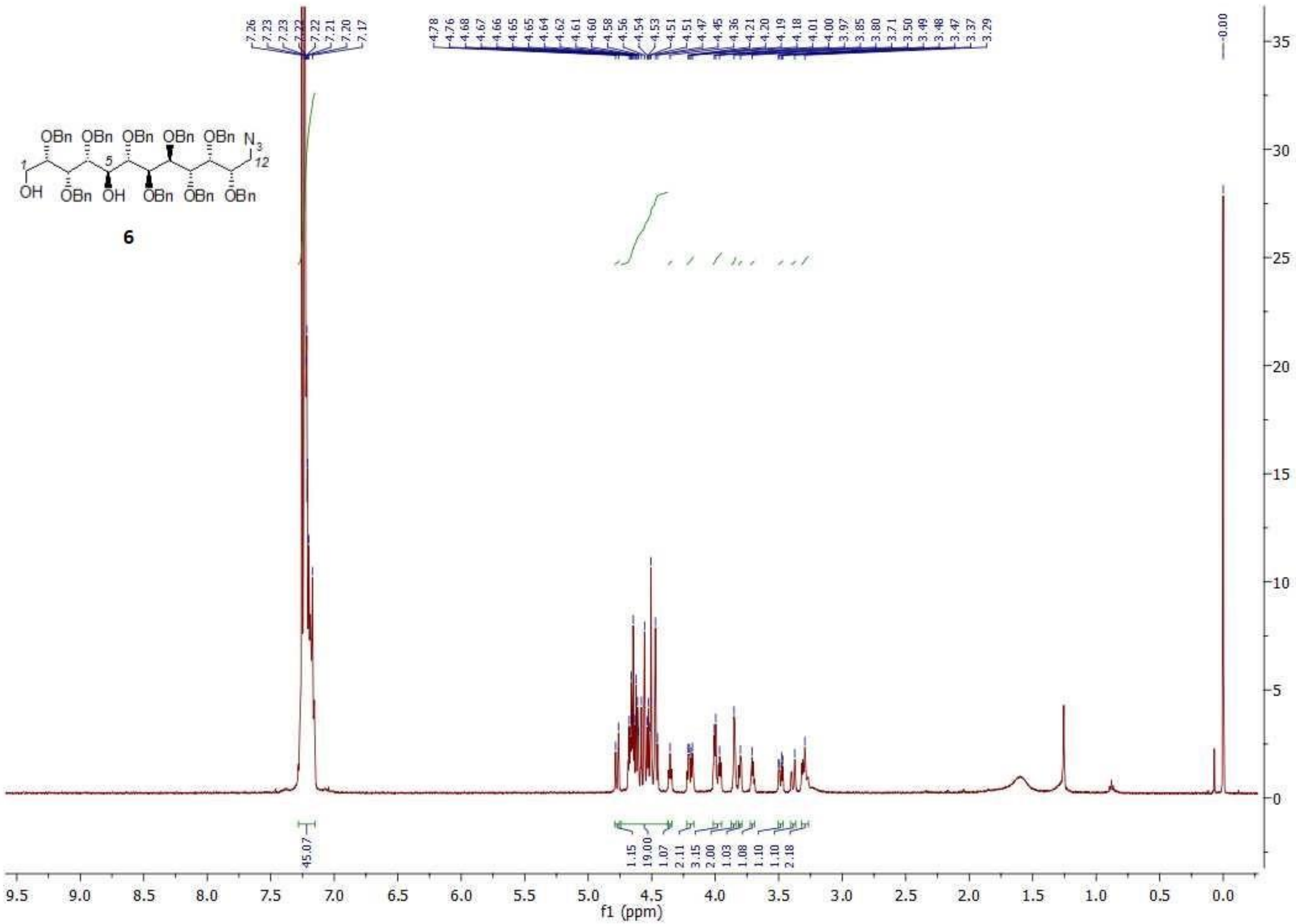

Figure S10: ^{1}H NMR spectrum of compound **6**.

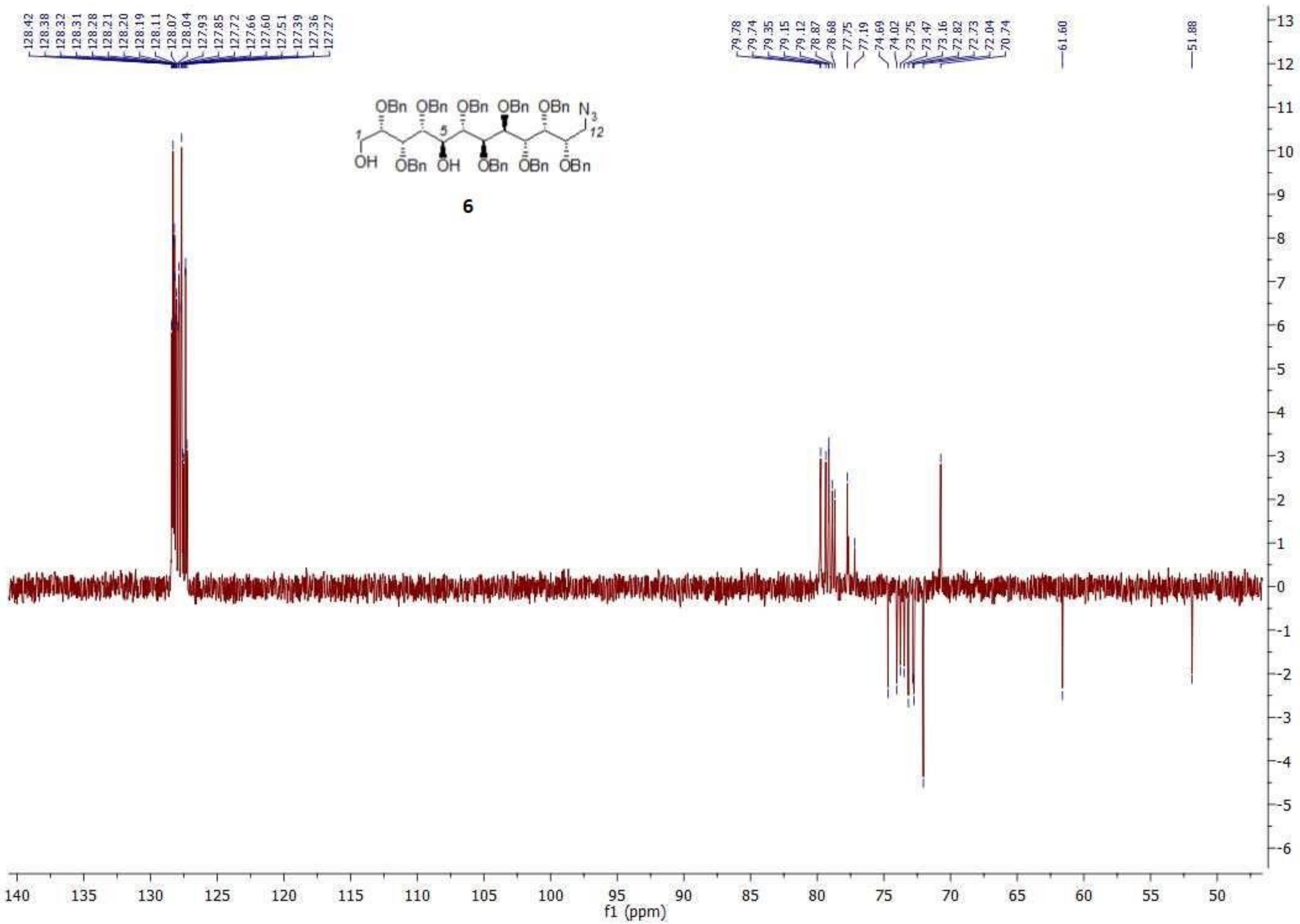

Figure S11: DEPT135 spectrum of compound **6**.

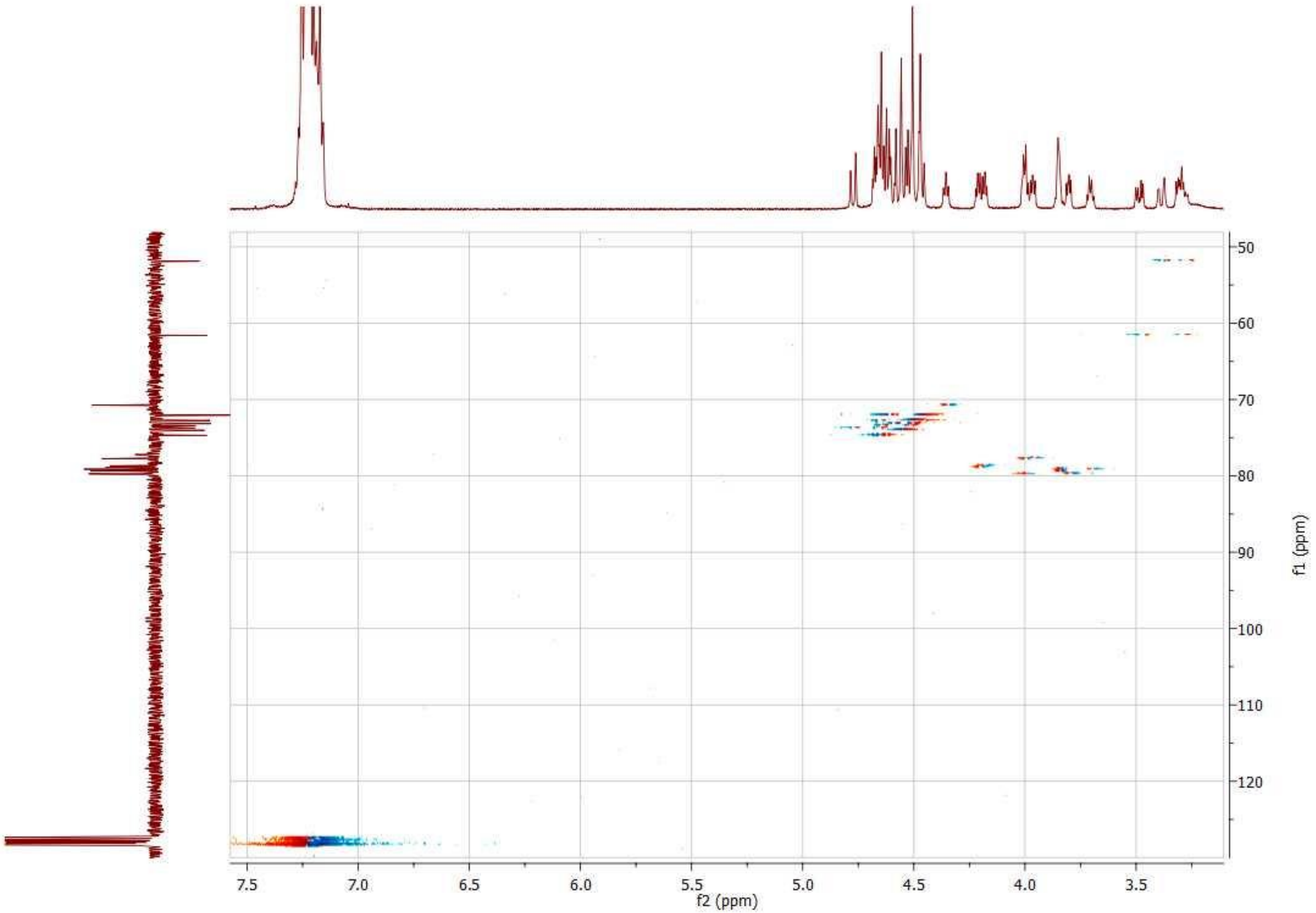

Figure S12: gHSQCAD spectrum of compound **6**.

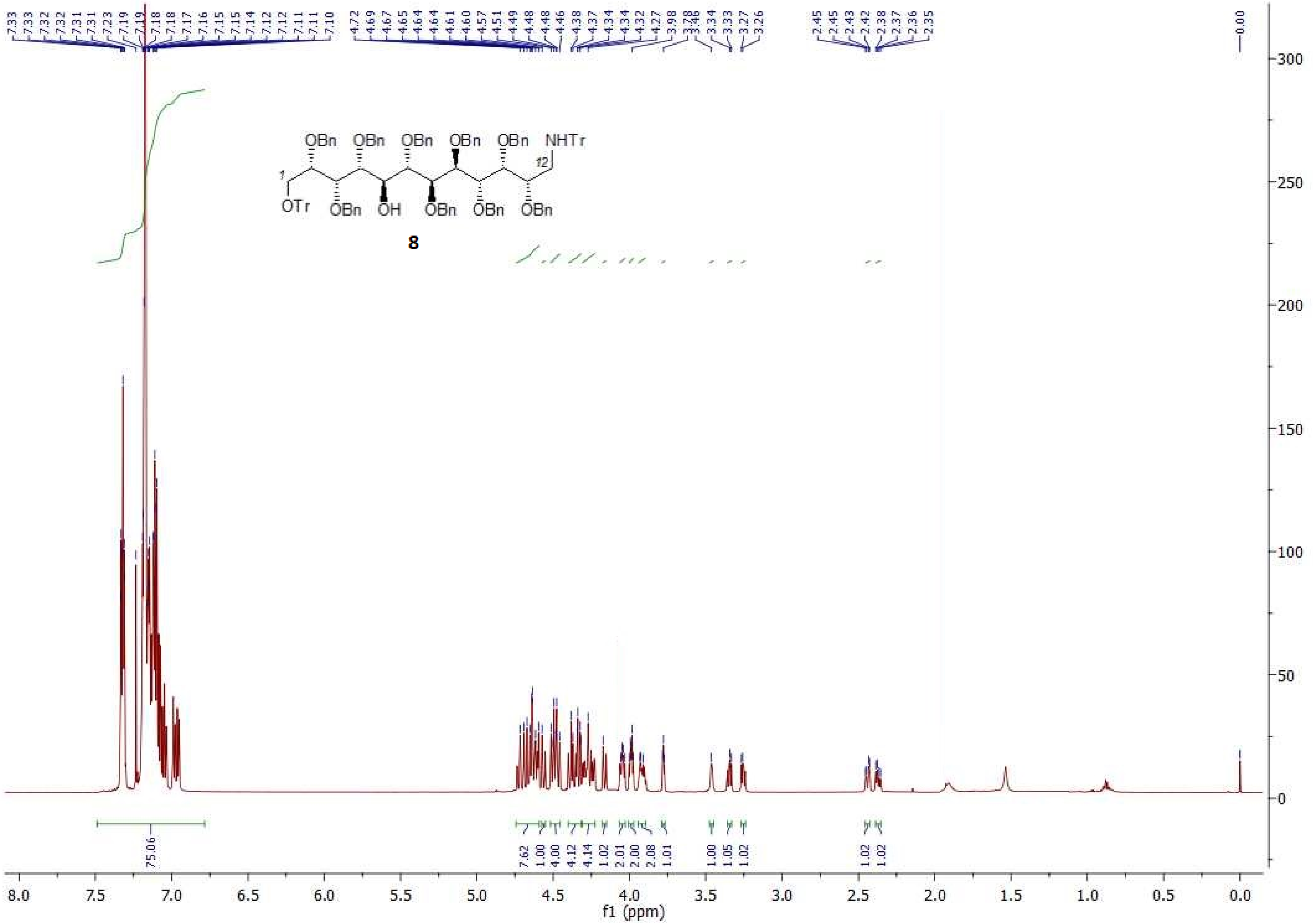

Figure S13: ^{1}H NMR spectrum of compound **8**.

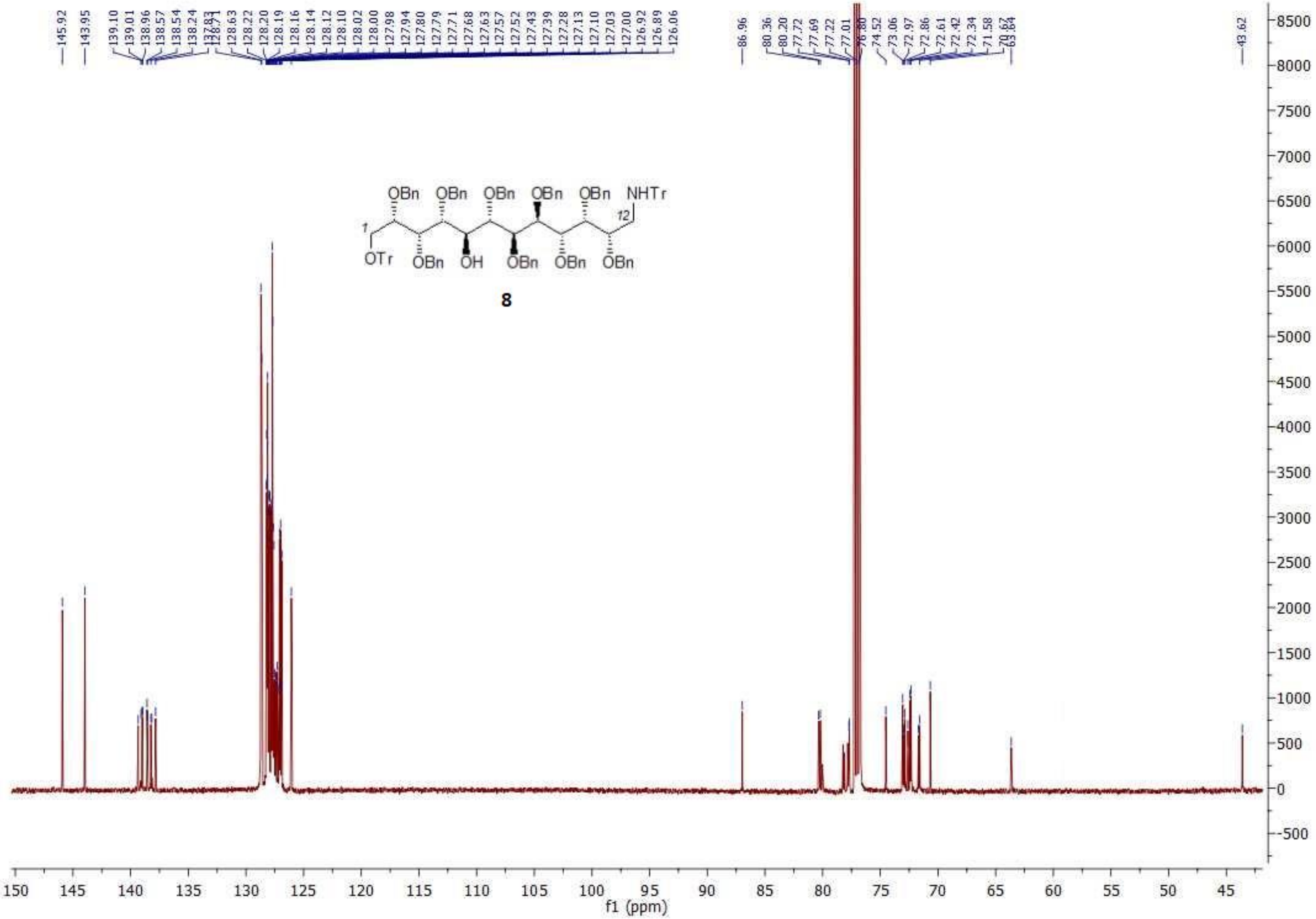

Figure S14: ^{13}C NMR spectrum of compound **8**.

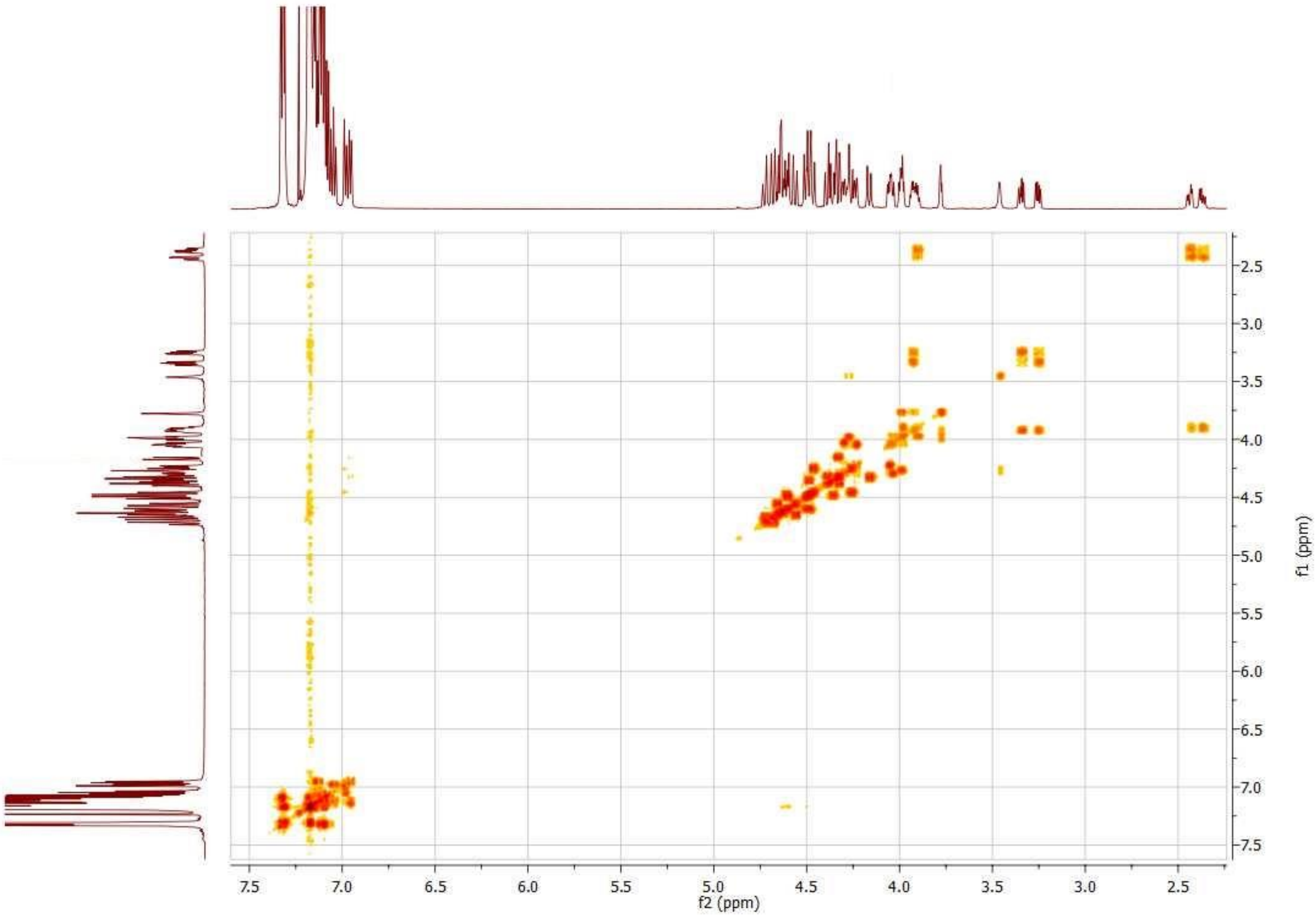

Figure S15: gCOSY spectrum of compound **8**.

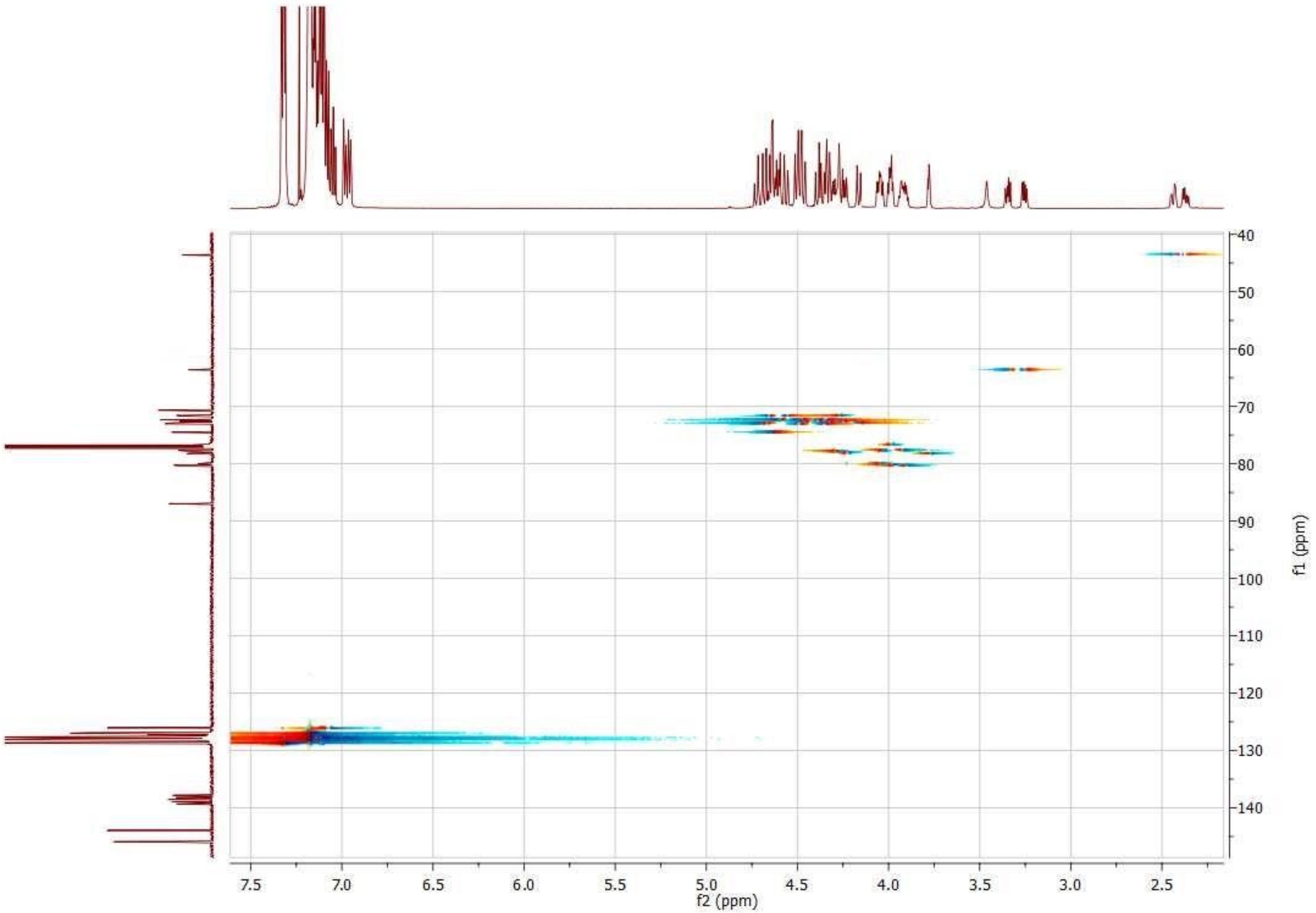

Figure S16: gHSQCAD spectrum of compound **8**.

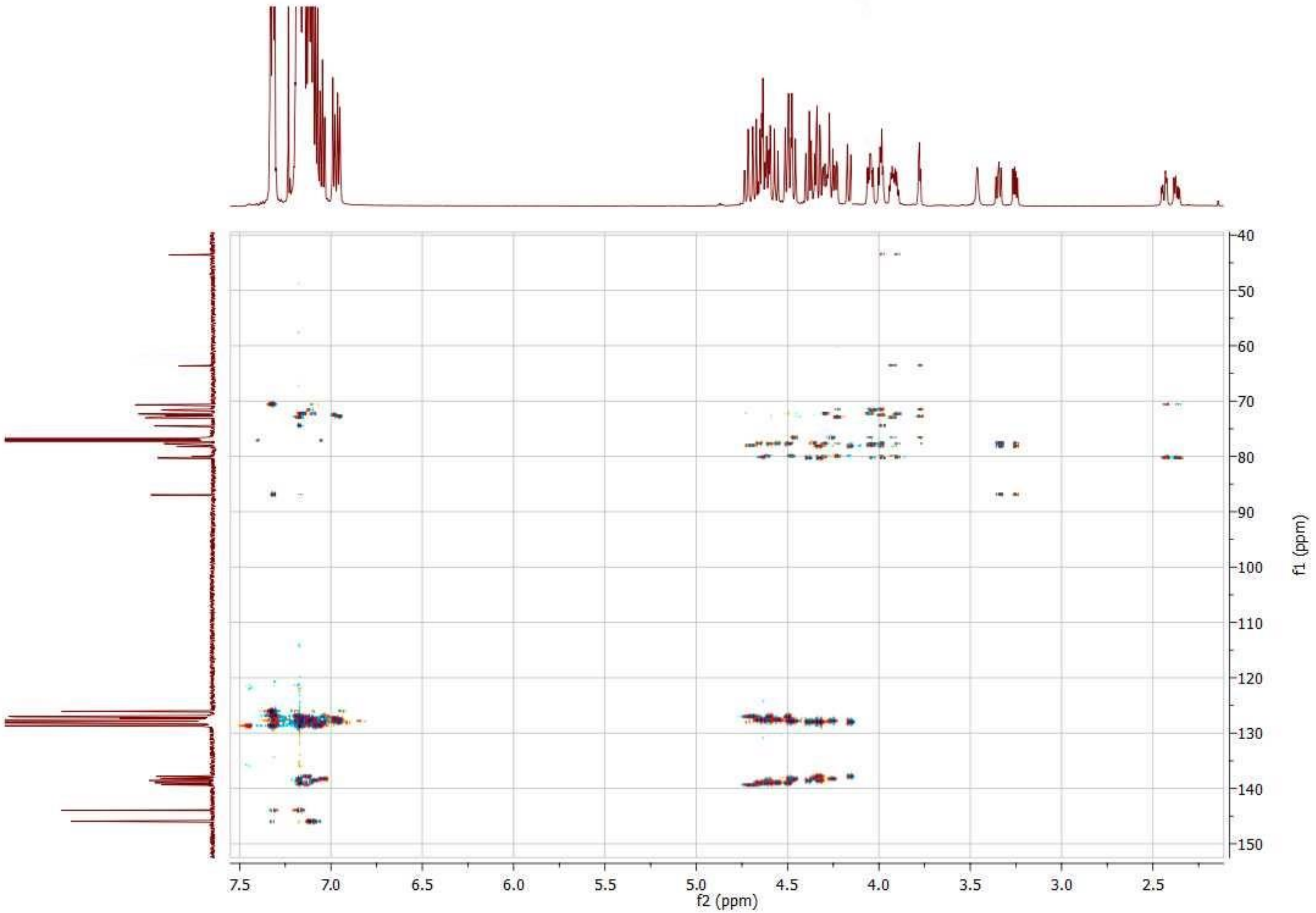

Figure S17: gHMBCAD spectrum of compound **8**.

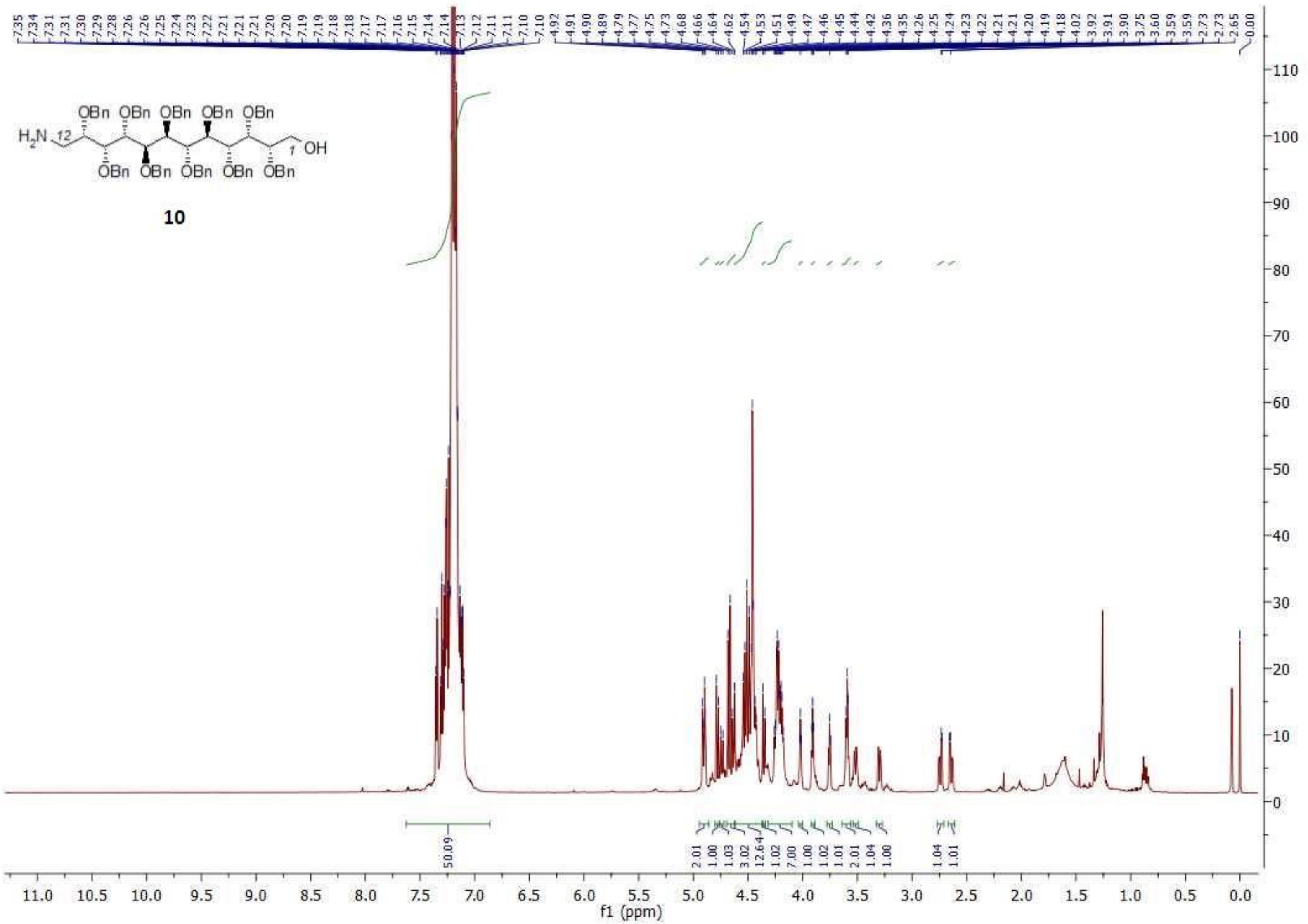

Figure S18: ^{1}H NMR spectrum of compound **10**.

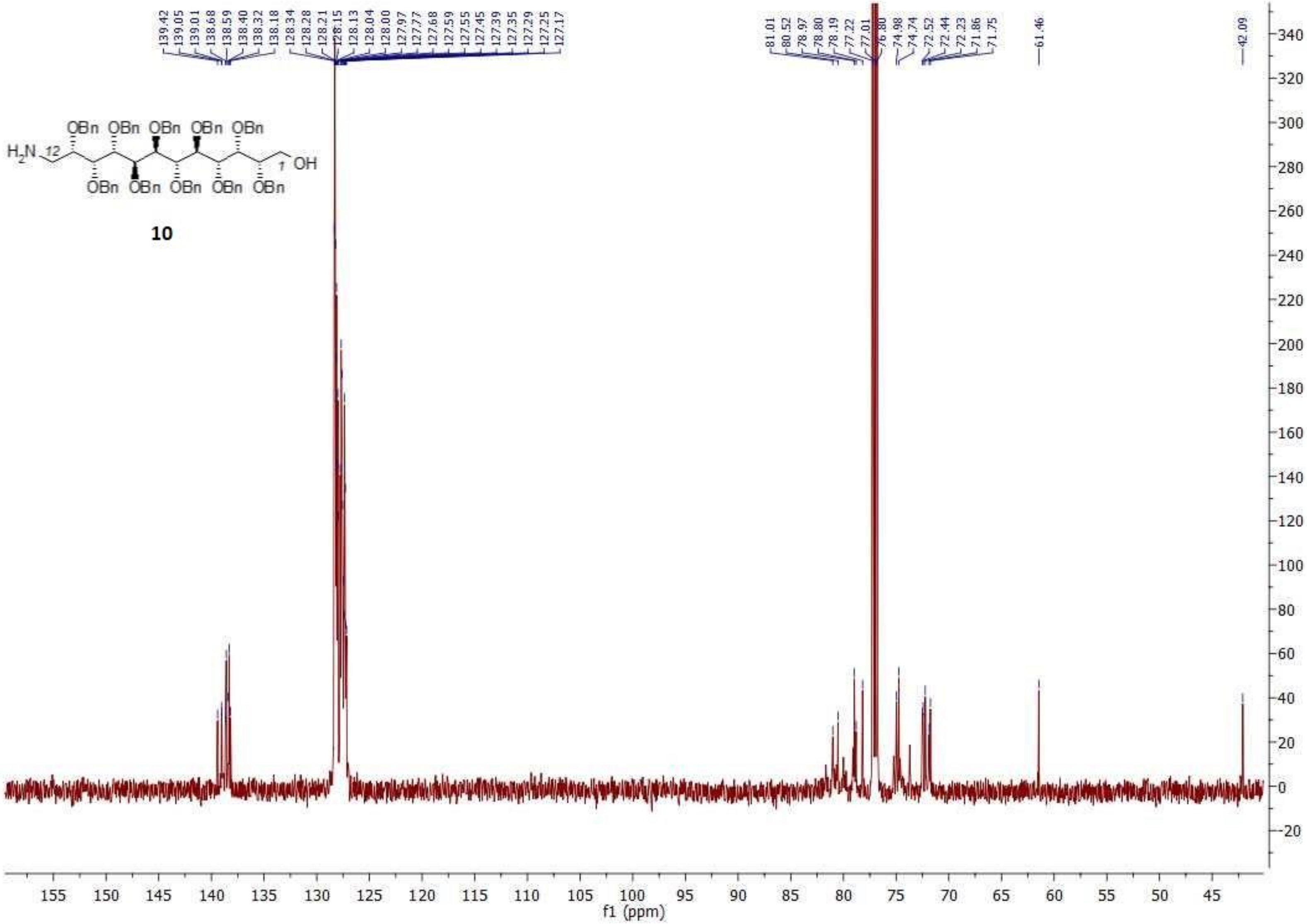

Figure S19: ^{13}C NMR spectrum of compound **10**.

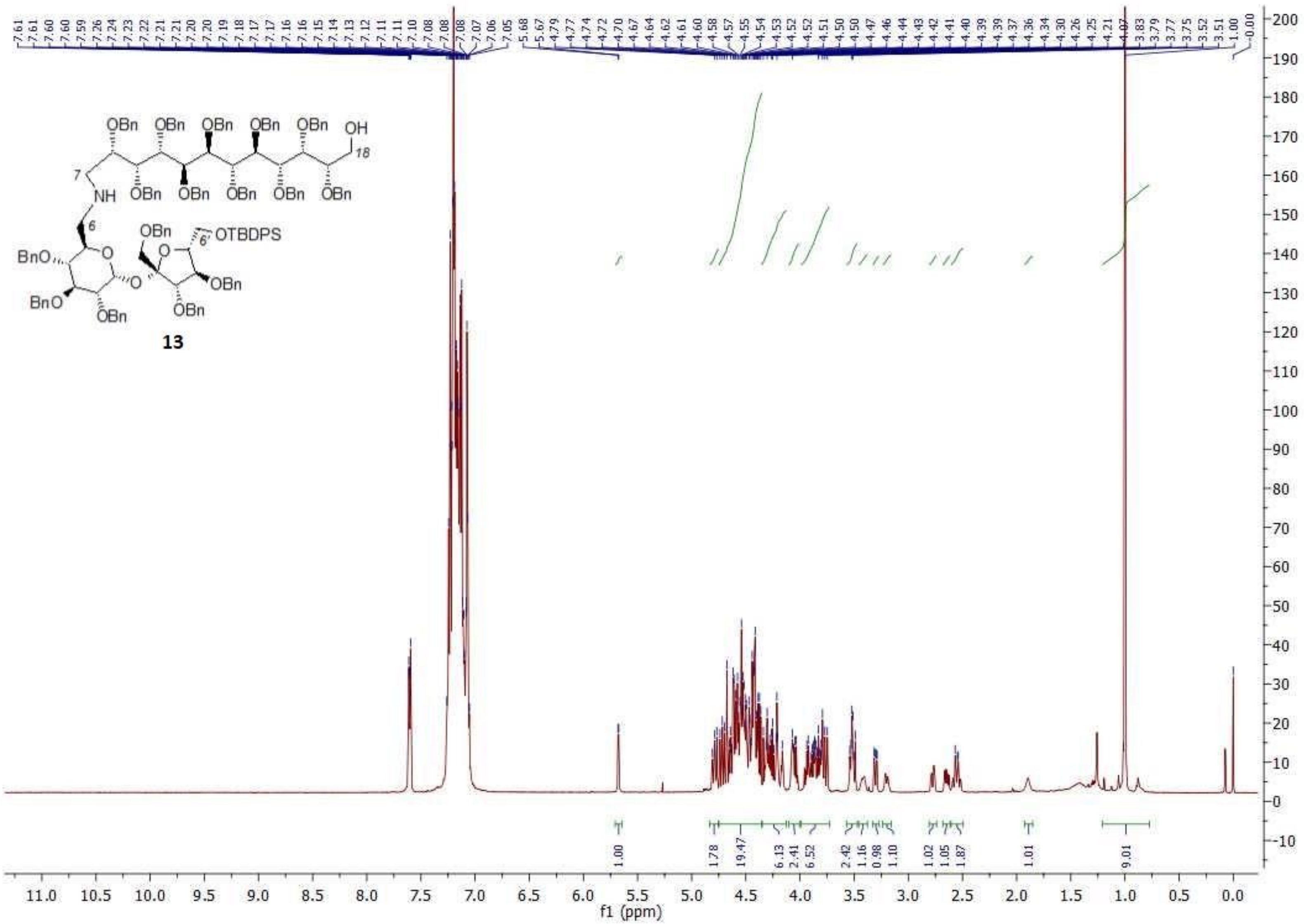

Figure S20: ^{1}H NMR spectrum of compound **13**.

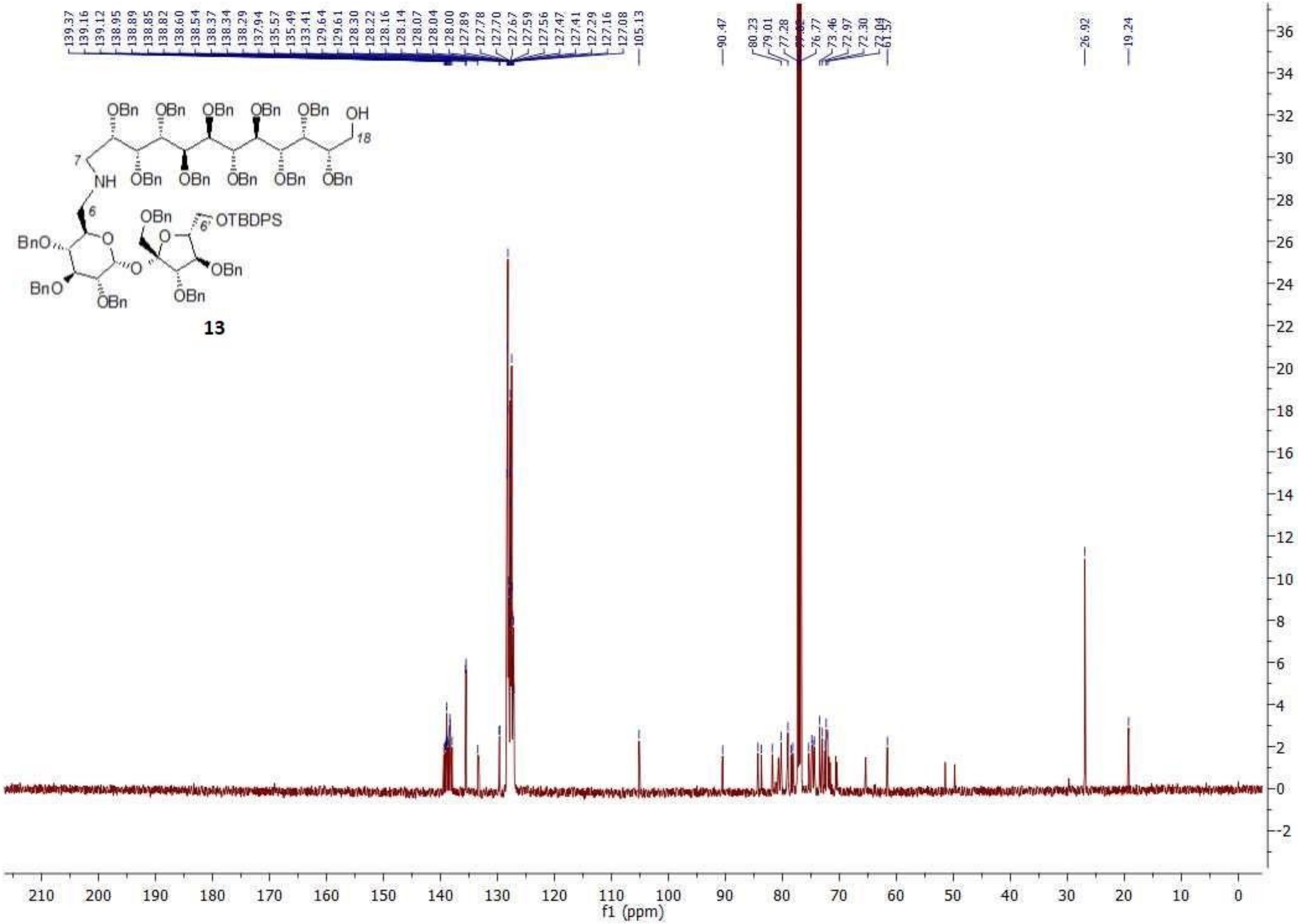

Figure S21: ^{13}C NMR spectrum of compound **13**.

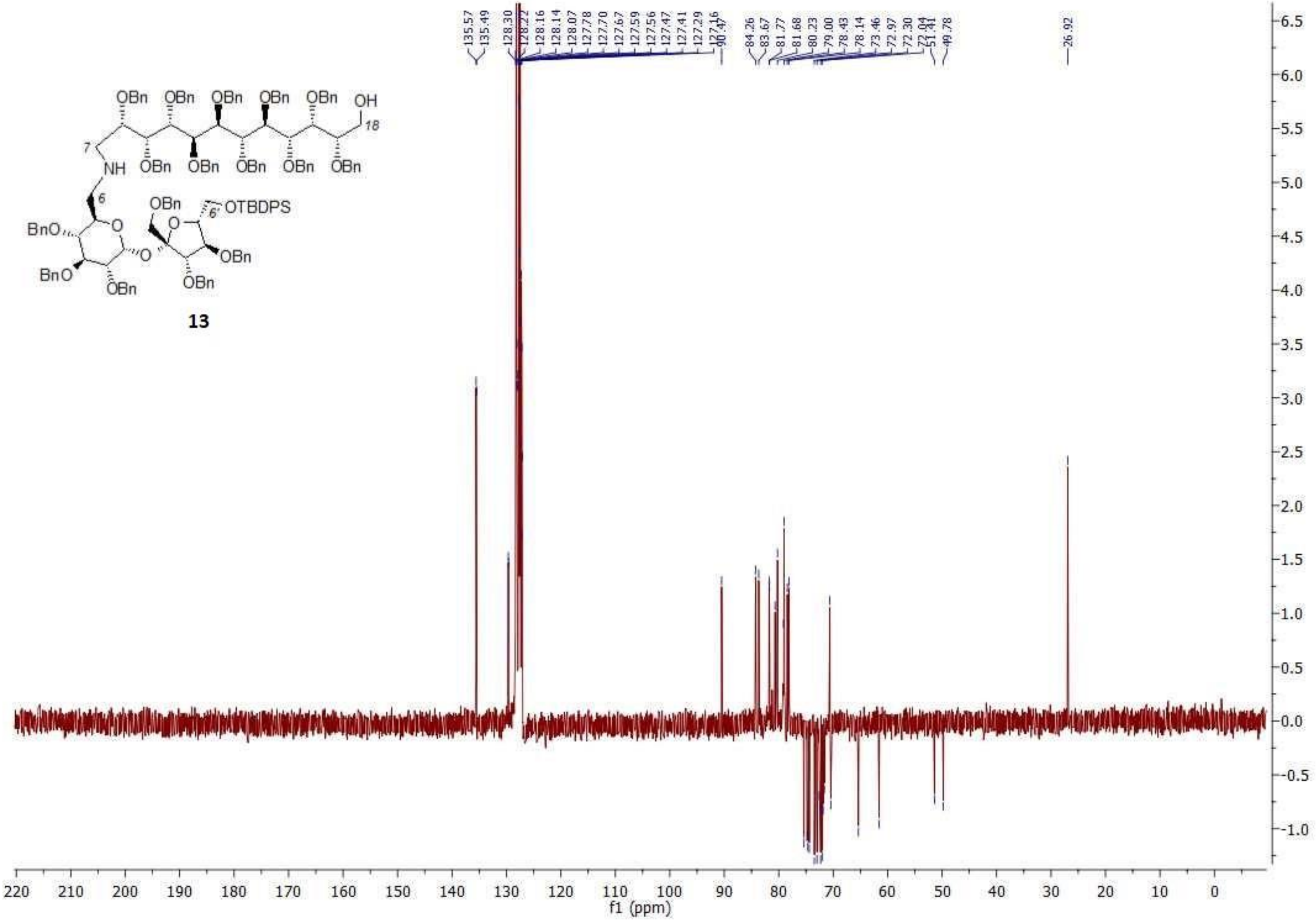

Figure S22: DEPT135 spectrum of compound **13**.

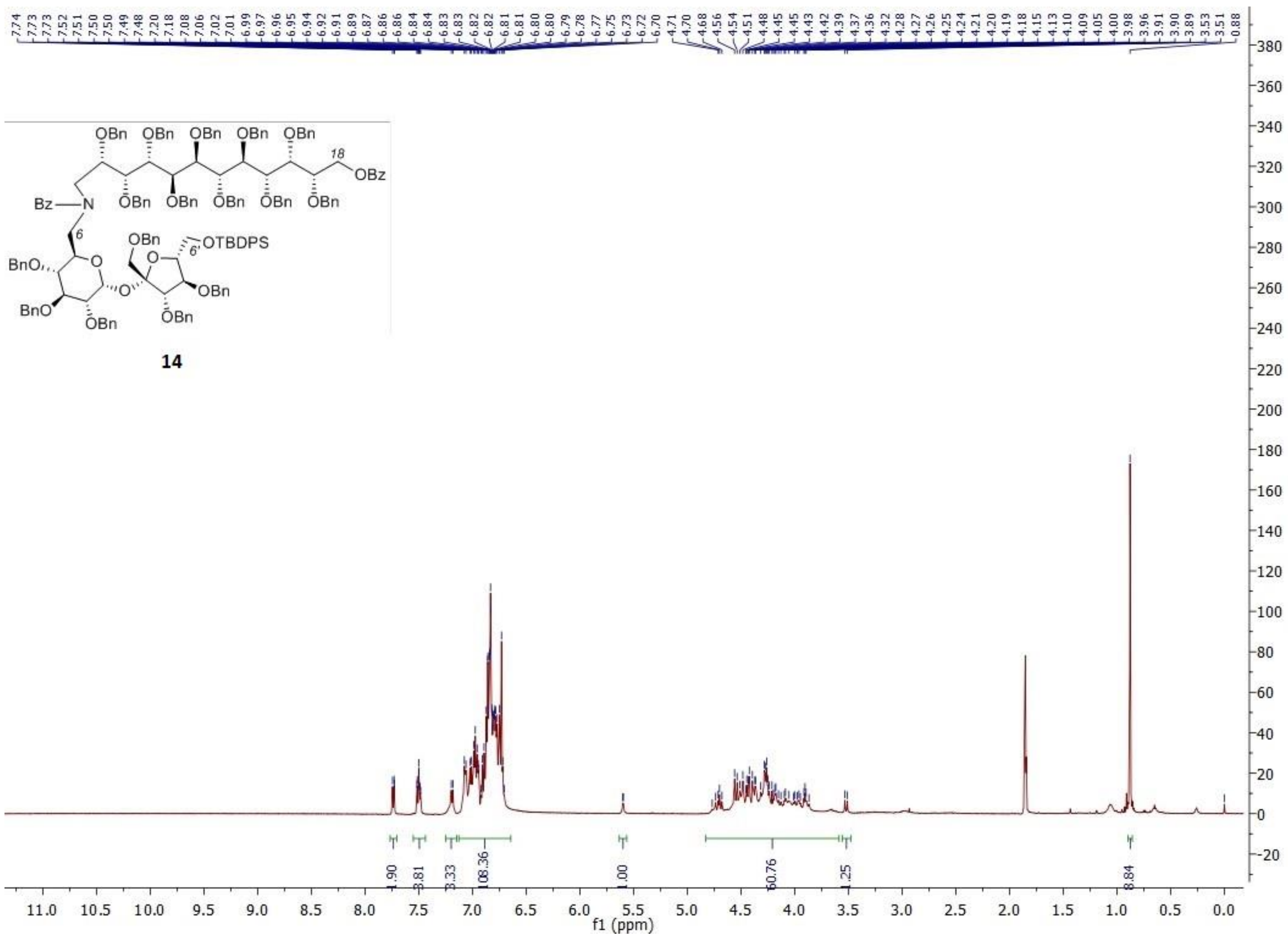

Figure S23: ^{1}H NMR spectrum of compound **14**.

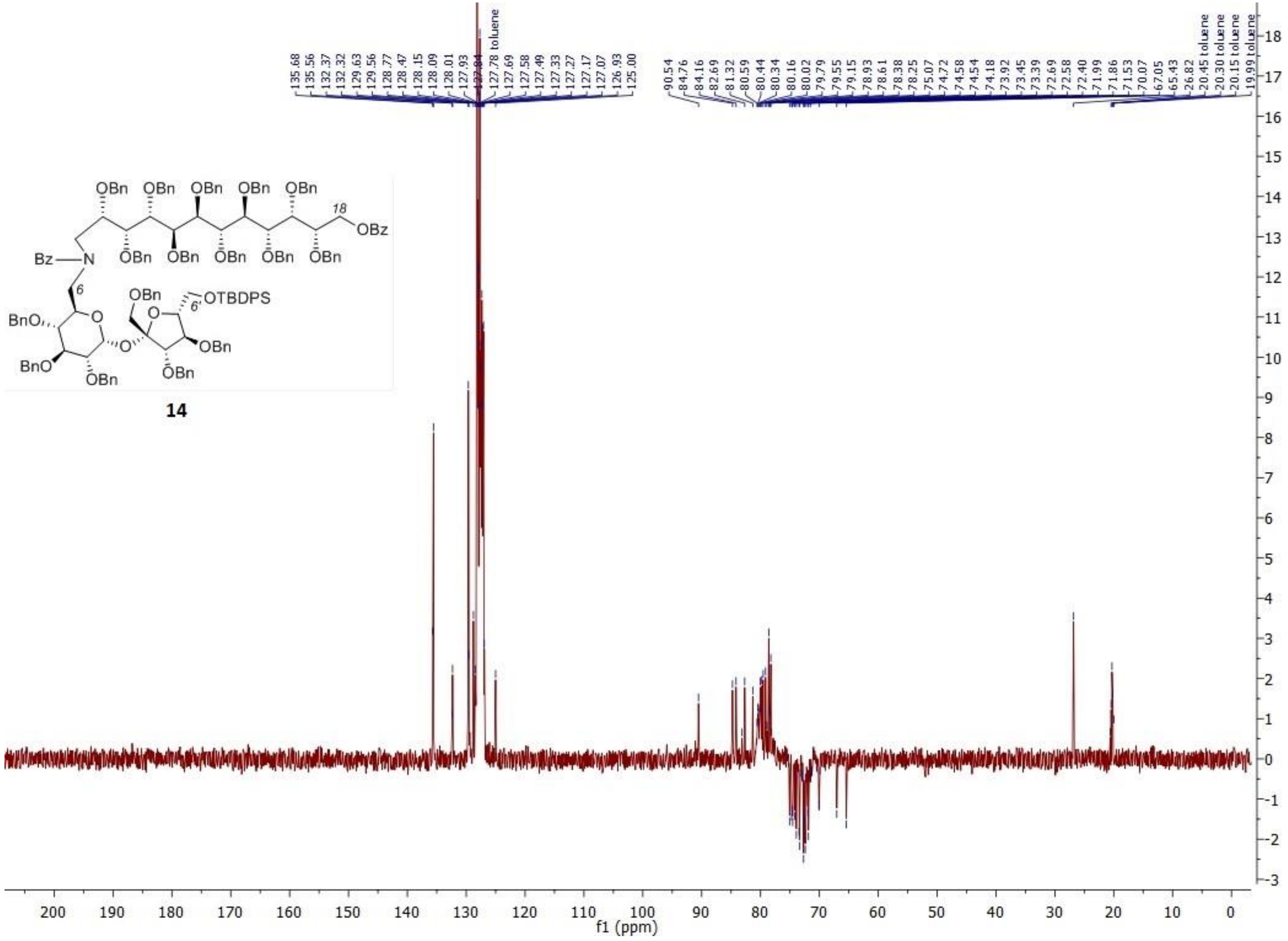

Figure S24: DEPT135 spectrum of compound **14**.

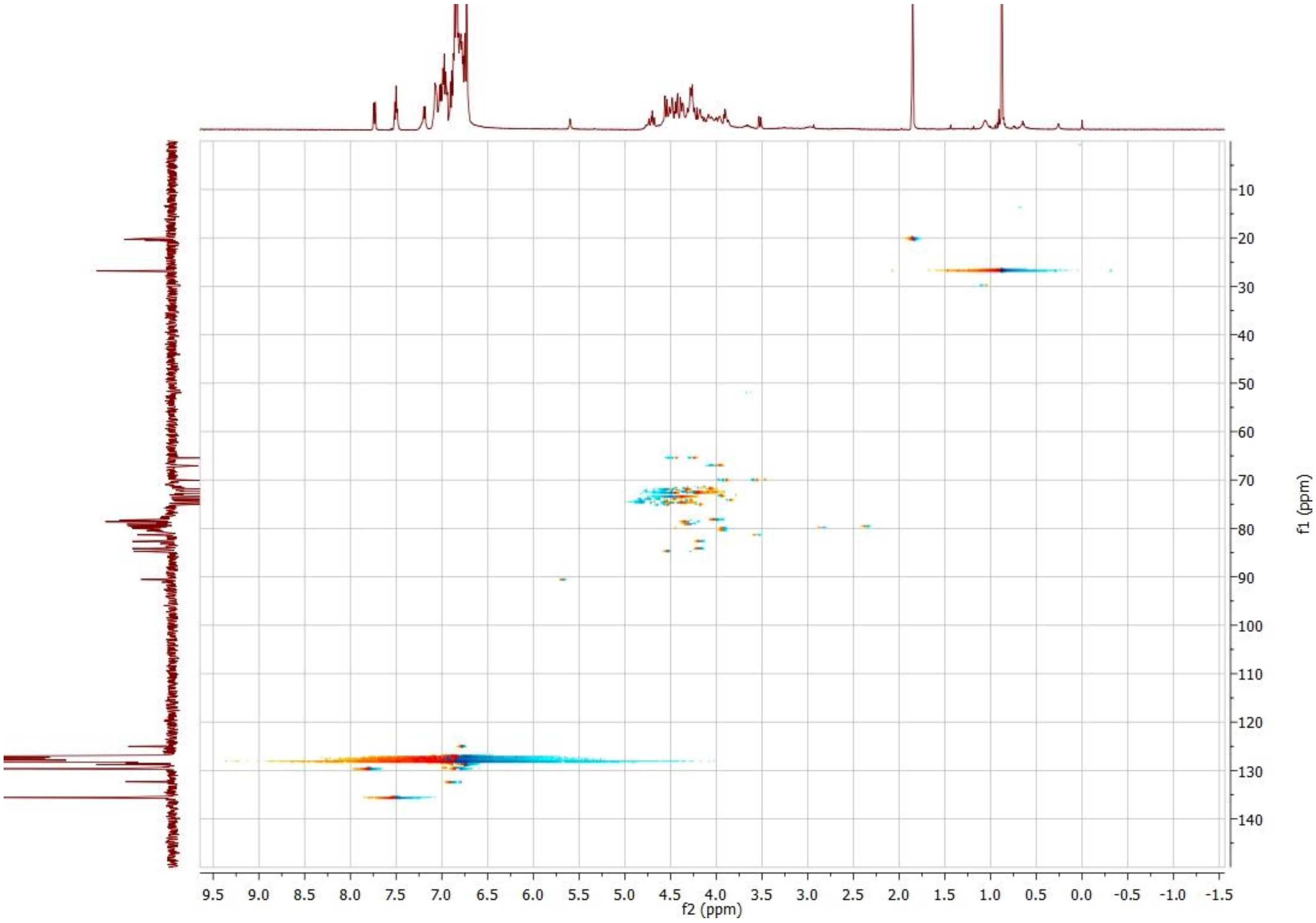

Figure S25: gHSQCAD spectrum of compound **14**.

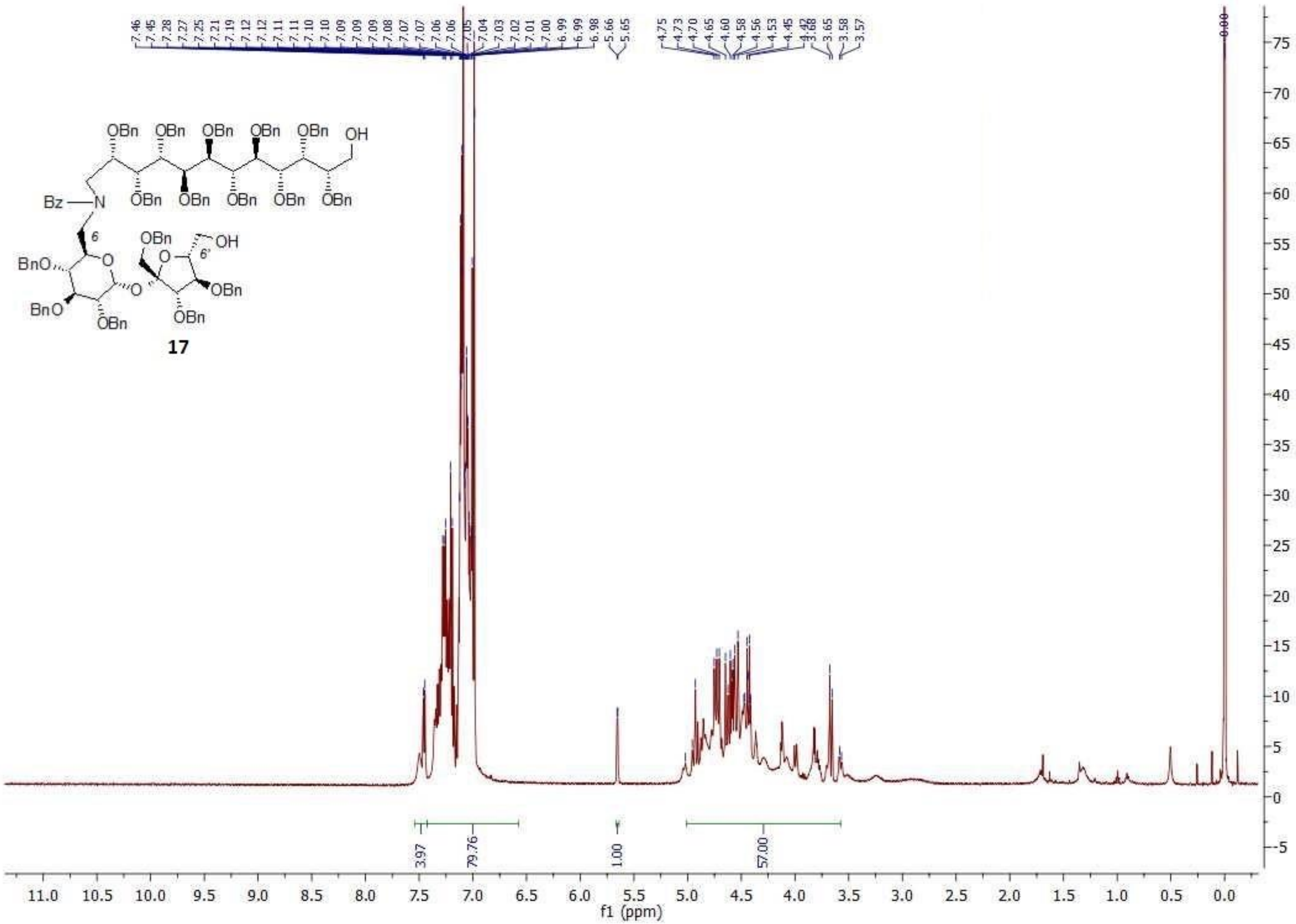

Figure S26: ^{1}H NMR spectrum of compound **17**.

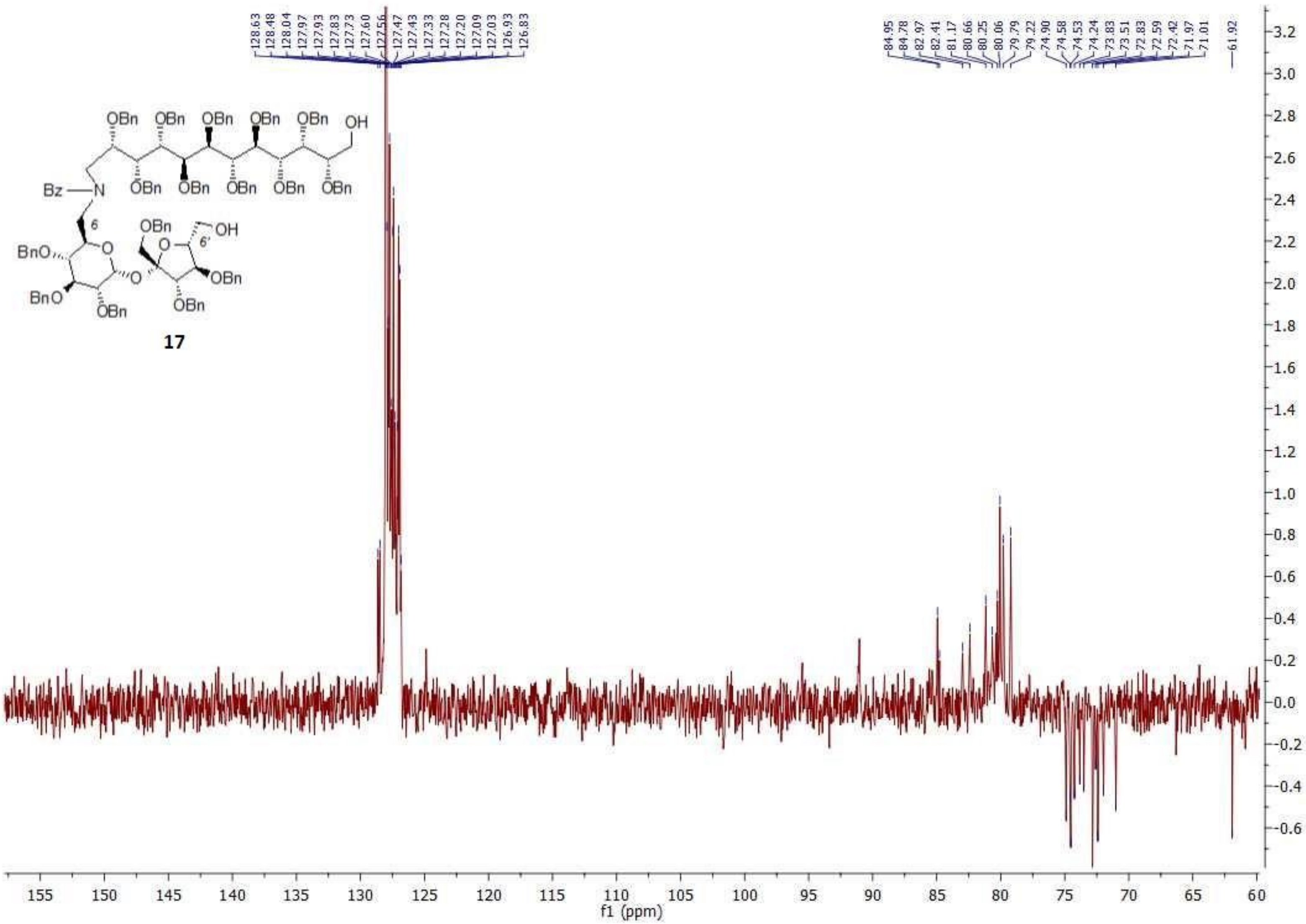

Figure S27: DEPT135 spectrum of compound **17**.
